# An Oxidant-Free and Mild Strategy for Quinazolin-4(3*H*)-One Synthesis via CuAAC/Ring Cleavage Reaction

**DOI:** 10.3390/molecules28155734

**Published:** 2023-07-28

**Authors:** Yueling He, Zhongtao Yang, Danyang Luo, Xiai Luo, Xiaodong Chen, Weiguang Yang

**Affiliations:** 1 The Marine Biomedical Research Institute of Guangdong Zhanjiang, Guangdong Medical University, Zhanjiang 524023, China; heyl1199122@163.com (Y.H.); yangzt@gdmu.edu.cn (Z.Y.); huluodanyang@163.com (D.L.); 2 School of Environmental Science and Engineering, Donghua University, Shanghai 201620, China; 3 Hunan Province Key Laboratory for Synthetic Biology of Traditional Chinese Medicine, School of Pharmaceutical Sciences, Hunan University of Medicine, Huaihua 418000, China; 4 Southern Marine Science and Engineering Guangdong Laboratory (Zhanjiang), Zhanjiang 524023, China

**Keywords:** CuAAC/ring cleavage reaction, nucleophilic addition, natural product, quinazolin-4(*3H*)-ones, ketenimine

## Abstract

An oxidant-free and highly efficient synthesis of phenolic quinazolin-4(3*H*)-ones was achieved by simply stirring a mixture of 2-aminobenzamides, sulfonyl azides, and terminal alkynes. The intermediate *N*-sulfonylketenimine underwent two nucleophilic additions and the sulfonyl group eliminated through the power of aromatization. The natural product 2-(4-hydroxybenzyl)quinazolin-4(*3H*)-one can be synthesized on a large scale under mild conditions with this method.

## 1. Introduction

**Figure 1:**
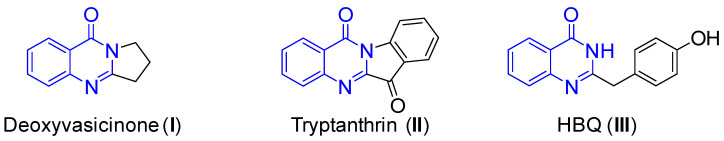
Natural products containing the quinazolin-4(3*H*)-one skeleton.

Due to their great physiological importance and pharmaceutical usefulness for fighting tumors, quinazolin-4(3*H*)-ones are promising compounds for biological and medicinal applications [1,2,3,4]. Some natural and synthetic quinazolin-4(3*H*)-ones with therapeutic properties are already being tested in clinical trials as potential drugs. For instance, natural products like deoxyvasicinone (**I**) [5] and tryptanthrin (**II**) [6,7,8] (Figure 1) have demonstrated antibacterial, antidepressant, and anti-inflammatory properties. The compound 2-(4-hydroxybenzyl) quinazolin-4(3*H*)-one (HBQ, **III**) [9,10], which is obtained from a fungus found in marine sediment, has been shown to have significant cytotoxic activity against certain cancer cell lines as well as strong inhibitory effects on the replication of tobacco mosaic virus (TMV). Given their versatile pharmacological and biological characteristics, there is always an urgent need for the synthesis of quinazolin-4(3*H*)-one products.

**Scheme 1:**
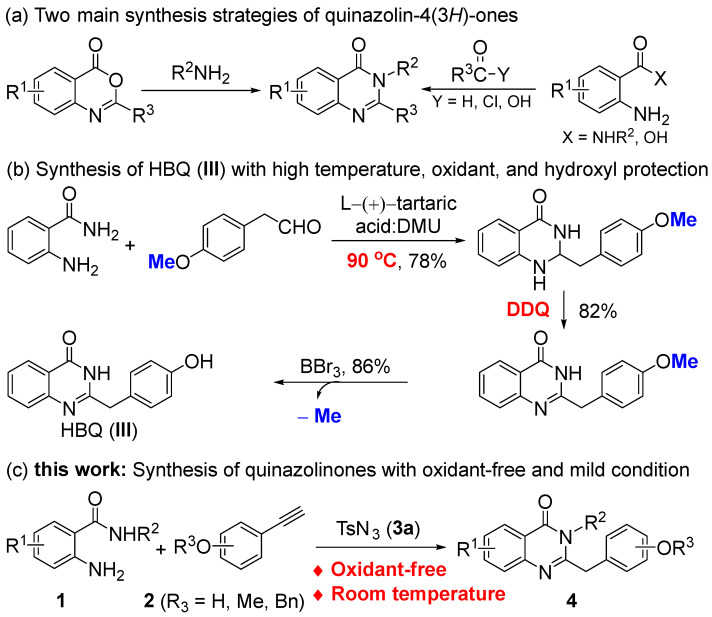
Strategies for the synthesis of quinazolin-4(3*H*)-ones.

Traditional methods for synthesizing quinazolin-4(3*H*)-ones involve two main approaches. One method is the amidation of benzoxazinones with arylamines [11,12]. The other method is the condensation of 2-aminobenzoyl derivatives with carbonyl derivatives (Scheme 1a). The latter method is the primary approach; the 2-aminobenzoyl derivatives included are 2-aminobenzamides [13,14,15,16,17,18,19,20,21,22,23,24], 2-aminobenzoic acid [11,25,26,27], 2-nitrobenzamides [28,29], and methyl anthranilate [30,31,32], while the carbonyl derivatives included are aldehydes [14,15], 1,3-diketones [16,17], orthoesters [18,19], benzyl alcohols [20,21,22], benzyl halides [23,24], acetophenones [33,34,35,36], methylarenes [37], and others [38,39]. Although most of these synthetic methods have their own merits, they often require extreme reaction conditions such as heating, using dehydration reagents, and adding an oxidant, which limits the synthesis of phenolic quinazolin-4(3*H*)-ones, or the phenol hydroxyl group needs to be protected in advance. For example, HBQ needs to be synthesized by oxidation from 4 (1*H*) quinazolinone using DDQ, which also requires high temperatures and protection of the phenol hydroxyl group from oxidation (Scheme 1b) [15].

Since it was reported by Chang’s group [40,41], the copper-catalyzed sulfonyl azide−alkyne cycloaddition/ring cleavage reaction (CuAAC/ring cleavage reaction) has been acknowledged as a gentle and effective method for synthesizing various nitrogenated compounds. It has also been used for modifying the structure of natural products, drugs, and biological macromolecules [42,43,44]. Our group has delved into this area and utilized the CuAAC/ring cleavage reaction to synthesize pyridine derivates, fused heterocycles, coumarins, indoles, and other nitrogenated compounds [45,46,47,48,49].

Therefore, in this study, we present a highly efficient and oxidant-free approach to synthesize phenolic quinazolin-4(3*H*)-ones using the CuAAC/ring cleavage reaction (Scheme 1c). This method involves stirring a mixture of 2-aminobenzamides, sulfonyl azides, and terminal alkynes in the presence of a copper(I) catalyst under mild conditions.

## 2. Results

Our investigations began with an examination of the synthesis of the parent and previously unreported system 3-benzyl-2-(3-hydroxybenzyl)quinazolin-4(*3H*)-one **4a** via 2-amino-*N*-benzylbenzamide **1a**, 3-ethynylphenol **2a**, and tosyl azide **3a** (Table 1). After an initial screening using CuI as a catalyst with the additive Et_3_N in a variety of solvents, we found that the desired conversion was affected by different solvents (Table 1, entries 1−10). The results revealed that MeCN generated product **4a** in the highest yield of 85%, the other solvents gave comparable yields, and EtOH generated product **4a** with the lowest yield of 34%. Encouraged by these promising results, a variety of catalysts were then evaluated, as shown in Table 1 (entries 11–17). Among the copper catalysts used, Cu^I^ catalysts (Table 1, entries 11–12) exhibited higher catalytic reactivity than Cu^II^ catalysts (Table 1, entries 13–16), and Cu(OTf)_2_ (Table 1, entry 17) was the least efficient for this reaction. Additional screening revealed that the other additives used were less efficient than Et_3_N (Table 1, entries 18–20). It is worth noting that the other sulfonyl azides such as MsN_3_ or PhSO_2_N_3_ were also suitable for this reaction (Table 1, entry 21).

**Scheme 2:**
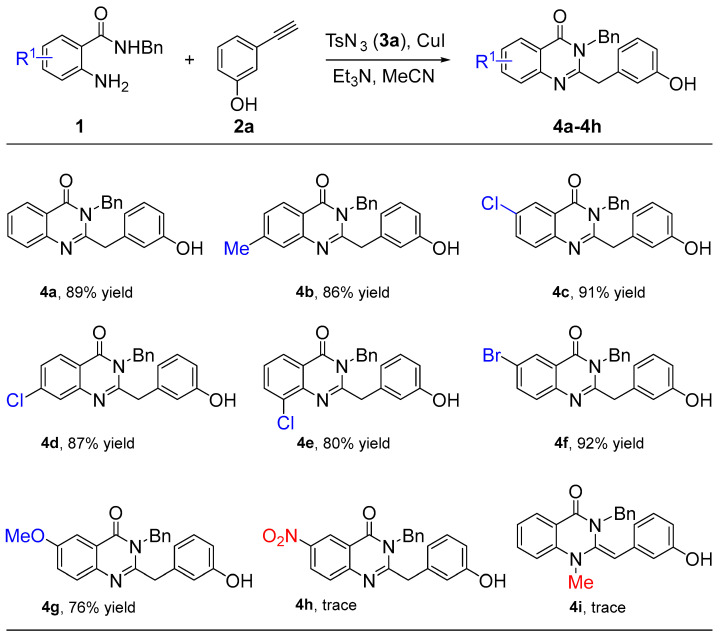
Substrate scope of 2-aminobenzamides **1**.

After the optimized reaction condition was established (Table 1, entry 5), the capacity of these reactions to affect the coupling of a range of different 2-aminobenzamides **1** was investigated. As shown in Scheme 2, the electronic effects of the substituents 2-aminobenzamides **1** had an obvious influence. For example, the substrate bearing a –Me group was examined, and an 82% yield of **4b** was isolated, which is the same efficiency as **4a**. When 2-aminobenzamides **1** carried halogen substituents including Cl or Br, the anticipated products (**4c**–**4f**) were also obtained in good yields ranging from 80% to 92%. However, the strong electron-donating substituent gave the corresponding product **4g** with a moderate yield of 76%, while the strongly electron-withdrawing substituent did not obtain the target product **4h** due to the weak nucleophilic activity of the amino group. Finally, when the –NH_2_ group was replaced by –NHMe, the target product **4i** was not obtained.

**Scheme 3:**
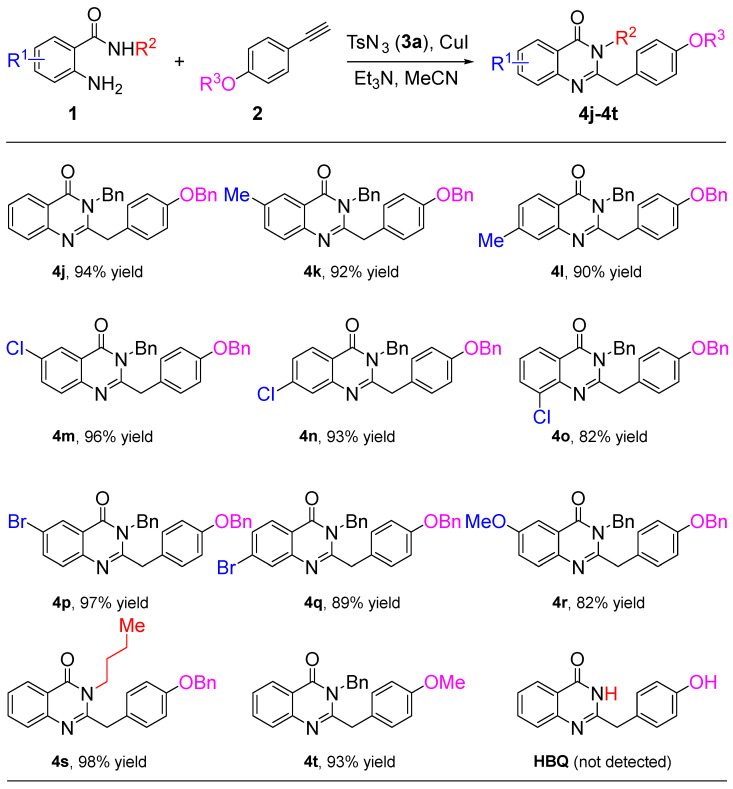
Substrate scope of 2-aminobenzamides **1** and terminal alkynes **2**.

The scope and limitations of different substrates with 2-aminobenzamides **1** and terminal alkynes **2** were also tested. As shown in Scheme 3, 2-aminobenzamides **1** exhibit the same electronic effect when 1-(benzyloxy)-4-ethynylbenzene is involved as a terminal alkyne in this reaction. The effect of the –Me group on the reaction is relatively small (**4j**–**4l**), the halogen groups are the most effective (**4m**–**4q**), and the strong electron-donating group is poor (**4r**). Expectedly, with R^2^ bearing an *^n^*-butyl or R^3^ bearing a –Me group, the corresponding quinazolin-4(*3H*)-one derivatives **4s** or **4t** are formed in an excellent yield of 98% and 93%, respectively. Disappointingly, the natural product 2-(4-hydroxybenzyl)quinazolin-4(*3H*)-one (HBQ, Figure 1, **III**) was not obtained when the R^2^ group changed to H of **1a**, which shows that the proton in this situation interferes with the reaction.

**Scheme 4:**
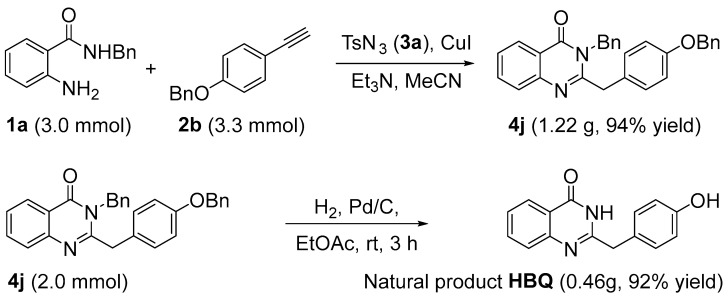
Synthesis of natural product HBQ.

Although the natural product HBQ cannot be directly obtained by the above method, it can be obtained by a simple reduction of product **4j** and can also be prepared in large quantities under mild conditions (Scheme 4).

**Scheme 5:**
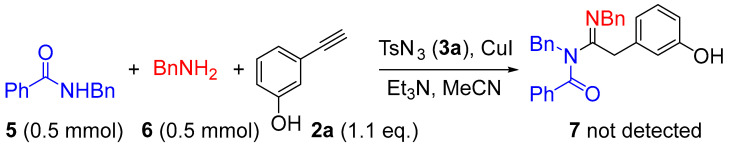
Control experiment.

What is interesting to us is that there was no sulfonyl group in the target products, and we could detect the other undesired product TsNH_2_, which we compared with standard samples by thin-layer chromatography (TLC) and confirmed by NMR. Moreover, unlike the other products, compound **4i**, which was difficult to synthesize (Scheme 2), was unaromatized. Therefore, we concluded that the product had aromatic properties. To confirm this fact and elucidate the mechanism, an intermolecular control experiment was performed under the optimized reaction condition (Table 1, entry 5). As shown in Scheme 5, *N*-Phenylbenzamide **5** and benzylamine **6** were tested for the intermolecular reaction. After being detected by TLC and confirmed by NMR, the *N*-sulfonylamidine product, which has been reported previously [41], was formed instead of the desired compound **7**. The above experiments show that aromaticity is indispensable.

**Scheme 6:**
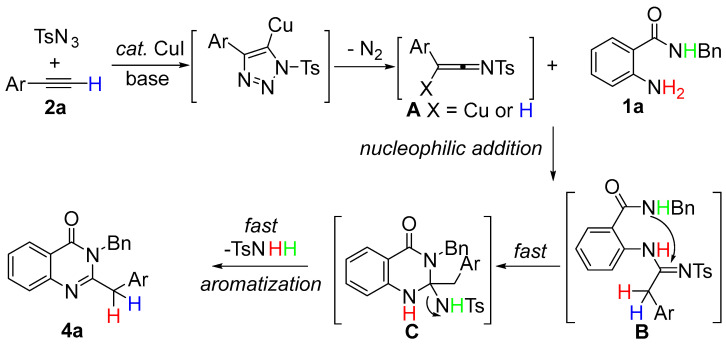
Plausible reaction mechanism.

Based on the above experiments, a possible reaction pathway for the synthesis of quinazolin-4(3*H*)-one **4a** was proposed (Scheme 6). According to the previous proposal [41,42,43,44,45,46,47,48,49,50], *N*-sulfonylketenimine **A** was generated first by the reaction of TsN_3_ and **2a**. Then, **A** underwent a nucleophilic addition reaction with **1a** to generate the intermediate **B**. Subsequently, intermediate **B** underwent an intramolecular cascade addition to generate the intermediate **C**. Lastly, the desired product **4a** and product TsNH_2_ were obtained by aromatization of intermediate **C**. We could not detect intermediates **B** and **C** during the experiment, which indicated that the procedure from **B** to **4a** was fast and almost simultaneous. The sulfonyl group was eliminated through the power of aromatization and activated the decomposition of the terminal alkynes into TsNH_2_ and N_2_.

## 3. Experimental Procedure

### 3.1. General Information

The ^1^H NMR spectra were recorded on a Bruker DPX 400 MHz spectrometer in CDCl_3_. Chemical shifts are reported in ppm with the internal TMS signal at 0.0 ppm as a standard. The spectra were interpreted as s, singlet; bs, broad singlet; d, doublet; t, triplet; q, quartet; m, multiplet; dd, double doublet; ddd, double double doublet; dt, double triplet; ddt, double double triplet; tt, triple triplet; td, triple doublet. Coupling constant(s) *J* are reported in Hz and relative integrations are reported. The ^13^C NMR (100 MHz) spectra were recorded on a Bruker DPX 400 MHz spectrometer in CDCl_3_. Chemical shifts are reported in ppm with the internal chloroform signal at 77.16 ppm as a standard and HBQ using CD_3_OD residual nondeuterated solvent as internal standard (CD_3_OD: *δ* 3.31 for ^1^H and 49.00 ppm for ^13^C). Melting points were obtained in open capillary tubes using the SGW X-4 micro melting point apparatus and were uncorrected. IR spectra were obtained with the Bruker Tensor-27 FT-IR spectrometer. Mass spectra were recorded on a TOF mass spectrometer. The starting materials, 2-amino-*N*-benzylbenzamide derivatives **1**, were all known and prepared according to the literature procedures [50,51]. Terminal alkynes **2**, TsN_3_ **3a**, and other reagents were purchased from Adamas-beta and other suppliers and used without further purification.

### 3.2. Compound Characterization and Preparations

At room temperature, to a solution of 2-amino-*N*-benzylbenzamides **1** (0.1 mmol, 1.0 equiv.), phenyl acetylenes **2** (0.11 mmol, 1.1 equiv.), CuI (1.9 mg, 10 mol%), TsN_3_ **3a** (21.7 mg, 0.11 mmol, 1.1 equiv.), and Et_3_N (11.1 mg, 0.11 mmol, 1.1 equiv.) in MeCN (2 mL) was added. The reaction mixture was stirred for 12 h. After completion of the reaction as indicated by TLC, the solvent was removed by evaporation in a vacuum. The residue was directly purified by flash column chromatography on silica gel (eluting with hexanes/EtOAc = 2:1) to form the corresponding product **4**. Some products contained the impurity sulfonamide which is difficult to separate when generated in this reaction.

*3-Benzyl-2-(3-hydroxybenzyl)quinazolin-4(3H)-one* (**4a**). White solid, 30.5 mg, yield: 89%, m.p: 180–182 °C. ^1^H NMR (400 MHz, CDCl_3_) *δ* 9.86 (s, 1H), 8.08–7.93 (m, 1H), 7.59–7.52 (m, 1H), 7.42 (dt, *J* = 9.6, 4.7 Hz, 2H), 7.36–7.20 (m, 4H), 7.10 (d, *J* = 7.2 Hz, 2H), 6.82 (d, *J* = 5.4 Hz, 2H), 6.74 (d, *J* = 7.7 Hz, 1H), 5.13 (s, 2H), 4.00 (s, 2H); ^13^C NMR (100 MHz, CDCl_3_) *δ* 161.8, 158.3, 156.8, 145.2, 135.8 (2C), 134.9, 131.0, 129.2 (2C), 128.0, 127.9, 127.5, 126.4 (2C), 124.9, 119.8, 119.7, 115.4, 113.6, 46.3, 41.5; IR *ν*_max_ (KBr): 3308, 2928, 1682, 1591, 1456, 1265, 1165, 976, 775, 731 cm^−1^; HRMS (ESITOF) *m*/*z* calcd for C_22H18_N_2_O_2_, [M + H]^+^ 343.1441, found 343.1443.
*3-Benzyl-2-(3-hydroxybenzyl)-7-methylquinazolin-4(3H)-one* (**4b**). White solid, 30.6 mg, yield: 86%, m.p: 184–186 °C. ^1^H NMR (400 MHz, CDCl_3_) *δ* 9.86 (s, 1H), 7.90 (dd, *J* = 8.5, 2.9 Hz, 1H), 7.38–7.28 (m, 3H), 7.27–7.20 (m, 2H), 7.15 (d, *J* = 2.5 Hz, 1H), 7.13–7.08 (m, 2H), 6.80 (d, *J* = 4.9 Hz, 2H), 6.73 (d, *J* = 7.6 Hz, 1H), 5.12 (s, 2H), 3.97 (s, 2H), 2.37 (s, 3H); ^13^C NMR (100 MHz, CDCl_3_) *δ* 161.7, 158.4, 156.8, 146.4, 145.4, 136.0, 135.9, 130.9, 129.9, 129.2, 129.0, 128.0, 127.8, 126.6, 126.4, 124.6, 119.7, 117.3, 115.3, 113.6, 46.0, 41.4, 22.1; IR *ν*_max_ (KBr): 3055, 1682, 1592, 1456, 1342, 1265, 1163, 974, 879, 737 cm^−1^; HRMS (ESITOF) *m*/*z* calcd for C_23H20_N_2_O_2_, [M + H]^+^ 357.1598, found 357.1599.
*3-Benzyl-6-chloro-2-(3-hydroxybenzyl)quinazolin-4(3H)-one* (**4c**). white solid, 34.2 mg, yield: 91%, m.p: 197–199 °C. ^1^H NMR (400 MHz, CDCl_3_) *δ* 9.23 (s, 1H), 8.11 (d, *J* = 2.6 Hz, 1H), 7.50 (dt, *J* = 8.3, 2.5 Hz, 1H), 7.37–7.21 (m, 5H), 7.10 (dd, *J* = 7.7, 3.2 Hz, 2H), 6.81 (d, *J* = 8.5 Hz, 1H), 6.78–6.72 (m, 2H), 5.15 (s, 2H), 4.00 (s, 2H); ^13^C NMR (100 MHz, CDCl_3_) *δ* 160.9, 158.1, 157.2, 143.9, 135.7, 135.5, 135.4, 133.5, 131.1, 129.3 (2C), 128.2, 127.0, 126.7, 126.5 (2C), 120.9, 120.0, 115.5, 113.5, 46.5, 41.5; IR *ν*_max_ (KBr): 3034, 2947, 1688, 1587, 1473, 1277, 1155, 980, 764, 717 cm^−1^; HRMS (ESITOF) *m*/*z* calcd for C_22H17_ClN_2_O_2_, [M + H]^+^ 377.1051, found 377.1056.
*3-Benzyl-7-chloro-2-(3-hydroxybenzyl)quinazolin-4(3H)-one* (**4d**). white solid, 32.7 mg, yield: 87%, m.p: 190–192 °C. ^1^H NMR (400 MHz, CDCl_3_) *δ* 8.23 (s, 1H), 8.14–8.03 (m, 1H), 7.47 (d, *J* = 2.7 Hz, 1H), 7.44–7.38 (m, 1H), 7.37–7.29 (m, 3H), 7.28–7.22 (m, 1H), 7.13 (d, *J* = 7.1 Hz, 2H), 6.81 (d, *J* = 8.3 Hz, 1H), 6.75 (d, *J* = 15.3 Hz, 2H), 5.17 (s, 2H), 4.00 (s, 2H); ^13^C NMR (100 MHz, CDCl_3_) *δ* 161.3, 156.0, 157.7, 146.8, 141.3, 135.8, 135.7, 131.0, 129.3 (2C), 129.2, 128.1 (2C), 126.4 (2C), 125.3, 120.2, 118.5, 115.4, 113.9, 46.5, 41.7; IR *ν*_max_ (KBr): 2924, 1684, 1601, 1456, 1331, 1232, 1265, 1159, 974, 731 cm^−1^; HRMS (ESITOF) *m*/*z* calcd for C_22H17_ClN_2_O_2_, [M + H]^+^ 377.1051, found 377.1056.
*3-Benzyl-8-chloro-2-(3-hydroxybenzyl)quinazolin-4(3H)-one* (**4e**). white solid, 30.1 mg, yield: 80%, m.p: 197–199 °C. ^1^H NMR (400 MHz, CDCl_3_) *δ* 8.21 (dd, *J* = 8.1, 3.0 Hz, 1H), 7.75 (dd, *J* = 7.9, 3.0 Hz, 1H), 7.42–7.27 (m, 4H), 7.18 (td, *J* = 7.7, 7.2, 4.2 Hz, 1H), 7.13 (d, *J* = 7.5 Hz, 2H), 6.83 (s, 1H), 6.75 (t, *J* = 9.9 Hz, 2H), 5.98 (s, 1H), 5.25 (s, 2H), 4.09 (s, 2H); ^13^C NMR (100 MHz, CDCl_3_) *δ* 162.2, 156.9, 156.7, 143.9, 136.5, 135.8, 134.9, 131.4, 130.5, 129.2 (2C), 128.0, 127.1, 126.3 (2C), 126.2, 122.2, 120.5, 115.1, 114.8, 46.6, 41.9; IR *ν*_max_ (KBr): 3007, 1676, 1580, 1445, 1389, 1275, 1159, 980, 849, 764 cm^−1^; HRMS (ESITOF) *m*/*z* calcd for C_22H17_ClN_2_O_2_, [M + H]^+^ 377.1051, found 377.1056.
*3-Benzyl-6-bromo-2-(3-hydroxybenzyl)quinazolin-4(3H)-one* (**4f**). white solid, 38.6 mg, yield: 92%, m.p: 176–178 °C. ^1^H NMR (400 MHz, CDCl_3_) *δ* 9.33 (s, 1H), 8.30 (q, *J* = 2.2 Hz, 1H), 7.68–7.57 (m, 1H), 7.38–7.21 (m, 5H), 7.10 (d, *J* = 7.2 Hz, 2H), 6.81 (d, *J* = 8.4 Hz, 1H), 6.75 (d, *J* = 10.2 Hz, 2H), 5.15 (s, 2H), 4.00 (s, 2H); ^13^C NMR (100 MHz, CDCl_3_) *δ* 160.7, 158.2, 157.4, 144.2, 138.2, 135.6, 135.5, 131.1, 130.3, 129.3 (2C), 128.2, 126.7, 126.5 (2C), 121.3, 121.2, 120.0, 115.5, 113.4, 46.5, 41.5; IR *ν*_max_ (KBr): 3026, 1684, 1587, 1456, 1389, 1277, 1153, 966, 831, 750 cm^−1^; HRMS (ESITOF) *m*/*z* calcd for C_22H17_BrN_2_O_2_, [M + H]^+^ 421.0546, found 421.0551.
*3-Benzyl-2-(3-hydroxybenzyl)-6-methoxyquinazolin-4(3H)-one* (**4g**). White solid, 28.3 mg, yield: 76%, m.p: 183–185 °C. ^1^H NMR (400 MHz, CDCl_3_) *δ* 10.06 (s, 1H), 7.42 (q, *J* = 2.7 Hz, 1H), 7.37–7.19 (m, 5H), 7.11 (td, *J* = 5.6, 2.7 Hz, 3H), 6.81 (d, *J* = 2.8 Hz, 2H), 6.73 (d, *J* = 7.6 Hz, 1H), 5.13 (s, 2H), 3.99 (s, 2H), 3.97 (s, 3H); ^13^C NMR (100 MHz, CDCl_3_) *δ* 161.7, 158.5, 158.4, 154.4, 139.6, 136.1, 136.0, 130.9, 129.8, 129.2, 128.0, 126.6, 126.4 (2C), 124.1, 120.8, 119.7, 115.4, 113.6, 108.1, 55.8, 46.4, 41.2; IR *ν*_max_ (KBr): 3005, 1670, 1593, 1495, 1456, 1362, 1275, 1155, 1028, 750 cm^−1^; HRMS (ESITOF) *m*/*z* calcd for C_23H20_N_2_O_3_, [M + H]^+^ 373.1547, found 373.1549.
*3-Benzyl-2-(4-(benzyloxy)benzyl)quinazolin-4(3H)-one* (**4j**). Oil, 40.6 mg, yield: 94%. ^1^H NMR (400 MHz, CDCl_3_) *δ* 8.33 (dd, *J* = 8.1, 2.8 Hz, 1H), 7.82–7.70 (m, 2H), 7.50 (d, *J* = 8.1 Hz, 1H), 7.44–7.21 (m, 8H), 7.17–7.11 (m, 4H), 6.93 (dd, *J* = 7.8, 3.1 Hz, 2H), 5.26 (s, 2H), 5.04 (s, 2H), 4.02 (s, 2H); ^13^C NMR (100 MHz, CDCl_3_) *δ* 162.8, 158.2, 155.9, 147.4, 136.9, 136.3, 134.6, 129.3 (2C), 129.1 (2C), 128.7 (2C), 128.1, 127.7, 127.6 (2C), 127.5, 127.4, 127.3, 127.0, 126.3 (2C), 120.6, 115.6 (2C), 70.2, 46.3, 41.6; IR *ν*_max_ (KBr): 3032, 1672, 1591, 1508, 1454, 1240, 1172, 1013, 750, 694 cm^−1^; HRMS (ESITOF) *m*/*z* calcd for C_29_H_24_N_2_O_2_, [M + H]^+^ 433.1911, found 433.1910.
*3-Benzyl-2-(4-(benzyloxy)benzyl)-6-methylquinazolin-4(3H)-one* (**4k**). White solid, 41.1 mg, yield: 92%, m.p: 137–139 °C. ^1^H NMR (400 MHz, CDCl_3_) *δ* 8.12 (s, 1H), 7.64 (dd, *J* = 8.3, 2.8 Hz, 1H), 7.61–7.57 (m, 1H), 7.45–7.22 (m, 8H), 7.13 (d, *J* = 7.3 Hz, 4H), 6.92 (dd, *J* = 8.3, 3.0 Hz, 2H), 5.26 (s, 2H), 5.04 (s, 2H), 4.01 (s, 2H), 2.50 (s, 3H); ^13^C NMR (100 MHz, CDCl_3_) *δ* 162.9, 158.2, 155.0, 145.5, 137.2, 137.0, 136.4, 136.0, 129.2 (2C), 129.1 (2C), 128.7 (2C), 128.1, 127.7 (2C), 127.6 (2C), 127.2, 126.7, 126.3 (2C), 120.4, 115.6 (2C), 70.2, 46.2, 41.5, 21.5; IR *ν*_max_ (KBr): 3032, 1670, 1591, 1508, 1454, 1340, 1275, 1013, 831, 750 cm^−1^; HRMS (ESITOF) *m*/*z* calcd for C_30_H_26_N_2_O_2_, [M + H]^+^ 447.2067, found 447.2069.
*3-Benzyl-2-(4-(benzyloxy)benzyl)-7-methylquinazolin-4(3H)-one* (**4l**). Oil, 40.2 mg, yield: 90%. ^1^H NMR (400 MHz, CDCl_3_) *δ* 8.21 (dd, *J* = 8.5, 3.0 Hz, 1H), 7.54 (s, 1H), 7.44–7.23 (m, 9H), 7.13 (d, *J* = 7.2 Hz, 4H), 6.95–6.89 (m, 2H), 5.25 (s, 2H), 5.03 (s, 2H), 4.00 (s, 2H), 2.51 (s, 3H); ^13^C NMR (100 MHz, CDCl_3_) *δ* 162.8, 158.2, 155.9, 147.5, 145.6, 137.0, 136.5, 129.3 (2C), 129.1 (2C), 128.7 (2C), 128.5, 128.1, 127.7, 127.6, 127.5 (2C), 127.1, 127.0, 126.3 (2C), 118.2, 115.6 (2C), 70.2, 46.1, 41.6, 22.0; IR *ν*_max_ (KBr): 3032, 1672, 1593, 1508, 1454, 1259, 1173, 1011, 750, 696 cm^−1^; HRMS (ESITOF) *m*/*z* calcd for C_30_H_26_N_2_O_2_, [M + H]^+^ 447.2067, found 447.2069.
*3-Benzyl-2-(4-(benzyloxy)benzyl)-6-chloroquinazolin-4(3H)-one* (**4m**). White solid, 44.7 mg, yield: 96%, m.p: 174–176 °C. ^1^H NMR (400 MHz, CDCl_3_) *δ* 8.20 (s, 1H), 7.66–7.55 (m, 2H), 7.38–7.15 (m, 8H), 7.04 (dd, *J* = 8.1, 2.9 Hz, 4H), 6.85 (dd, *J* = 8.7, 3.0 Hz, 2H), 5.17 (s, 2H), 4.96 (s, 2H), 3.93 (s, 2H); ^13^C NMR (100 MHz, CDCl_3_) *δ* 161.9, 158.3, 156.2, 146.0, 136.9, 136.0, 135.0, 132.7, 129.3 (2C), 129.2 (2C), 129.1, 128.7 (2C), 128.2, 127.9, 127.6 (2C), 127.2, 126.6, 126.3 (2C), 121.7, 115.6 (2C), 70.2, 46.4, 41.5; IR *ν*_max_ (KBr): 3034, 1676, 1591, 1508, 1472, 1335, 1275, 1013, 835, 750 cm^−1^; HRMS (ESITOF) *m*/*z* calcd for C_29_H_23_ClN_2_O_2_, [M + H]^+^ 467.1521, found 467.1528.
*3-Benzyl-2-(4-(benzyloxy)benzyl)-7-chloroquinazolin-4(3H)-one* (**4n**). Oil, 43.4 mg, yield: 93%. ^1^H NMR (400 MHz, CDCl_3_) *δ* 8.24 (dd, *J* = 9.0, 3.1 Hz, 1H), 7.73 (d, *J* = 3.0 Hz, 1H), 7.47–7.23 (m, 9H), 7.13 (d, *J* = 6.8 Hz, 4H), 6.93 (dd, *J* = 8.1, 3.2 Hz, 2H), 5.25 (s, 2H), 5.04 (s, 2H), 4.00 (s, 2H); ^13^C NMR (100 MHz, CDCl_3_) *δ* 162.3, 158.3, 157.3, 148.4, 140.8, 136.9, 136.1, 129.3 (2C), 129.2 (2C), 128.8, 128.7 (2C), 128.2, 127.9, 127.6 (2C), 127.5, 127.2, 127.0, 126.3 (2C), 119.1, 115.6 (2C), 70.2, 46.3, 41.5; IR *ν*_max_ (KBr): 3034, 1676, 1591, 1508, 1454, 1383, 1240, 1013, 748, 694 cm^−1^; HRMS (ESITOF) *m*/*z* calcd for C_29_H_23_ClN_2_O_2_, [M + H]^+^ 467.1521, found 467.1528.
*3-Benzyl-2-(4-(benzyloxy)benzyl)-8-chloroquinazolin-4(3H)-one* (**4o**). White solid, 38.2 mg, yield: 82%, m.p: 111–113 °C. ^1^H NMR (400 MHz, CDCl_3_) *δ* 8.22 (dd, *J* = 8.0, 2.8 Hz, 1H), 7.84 (dd, *J* = 7.9, 2.8 Hz, 1H), 7.44–7.35 (m, 5H), 7.34–7.26 (m, 4H), 7.16 (d, *J* = 7.8 Hz, 2H), 7.12 (d, *J* = 6.7 Hz, 2H), 6.93 (dd, *J* = 8.0, 2.6 Hz, 2H), 5.26 (s, 2H), 5.04 (s, 2H), 4.07 (s, 2H); ^13^C NMR (100 MHz, CDCl_3_) *δ* 162.3, 158.3, 156.8, 144.2, 137.0, 135.9, 134.8, 129.5 (2C), 129.2 (2C), 128.91, 128.7 (2C), 128.1, 127.9, 127.6 (2C), 127.3, 126.9, 126.3 (2C), 126.1, 122.2, 115.6 (2C), 70.2, 46.5, 41.7; IR *ν*_max_ (KBr): 3030, 1676, 1591, 1508, 1445, 1261, 1163, 987, 748, 696 cm^−1^; HRMS (ESITOF) *m*/*z* calcd for C_29_H_23_ClN_2_O_2_, [M + H]^+^ 467.1521, found 467.1528.
*3-Benzyl-2-(4-(benzyloxy)benzyl)-6-bromoquinazolin-4(3H)-one* (**4p**). White solid, 49.5 mg, yield: 97%, m.p: 167–169 °C. ^1^H NMR (400 MHz, CDCl_3_) *δ* 8.45 (t, *J* = 2.4 Hz, 1H), 7.83 (dd, *J* = 8.7, 2.6 Hz, 1H), 7.59 (dd, *J* = 9.4, 3.0 Hz, 1H), 7.44–7.29 (m, 8H), 7.12 (d, *J* = 6.8 Hz, 4H), 6.93 (dd, *J* = 7.8, 2.8 Hz, 2H), 5.25 (s, 2H), 5.04 (s, 2H), 4.00 (s, 2H); ^13^C NMR (100 MHz, CDCl_3_) *δ* 161.7, 158.3, 156.3, 146.3, 137.7, 136.9, 136.0, 129.8, 129.3 (2C), 129.2, 129.1 (2C), 128.7 (2C), 128.2, 127.9, 127.6 (2C), 127.2, 126.3 (2C), 122.0, 120.4, 115.6 (2C), 70.2, 46.4, 41.5; IR *ν*_max_ (KBr): 2905, 1676, 1589, 1510, 1467, 1333, 1275, 985, 750, 692 cm^−1^; HRMS (ESITOF) *m*/*z* calcd for C_29_H_23_BrN_2_O_2_, [M + H]^+^ 511.1016, found 511.1021.
*3-Benzyl-2-(4-(benzyloxy)benzyl)-7-bromoquinazolin-4(3H)-one* (**4q**). Oil, 45.4 mg, yield: 89%. ^1^H NMR (400 MHz, CDCl_3_) *δ* 8.16 (dd, *J* = 8.8, 3.0 Hz, 1H), 7.91 (s, 1H), 7.58 (dd, *J* = 8.6, 2.7 Hz, 1H), 7.45–7.23 (m, 8H), 7.13 (d, *J* = 6.9 Hz, 4H), 6.97–6.90 (m, 2H), 5.25 (s, 2H), 5.04 (s, 2H), 4.00 (s, 2H); ^13^C NMR (100 MHz, CDCl_3_) *δ* 162.4, 158.3, 157.3, 148.4, 136.9, 136.0, 130.3, 130.2, 129.4 (2C), 129.3, 129.2 (2C), 128.8, 128.7 (2C), 128.2, 127.9, 127.6 (2C), 127.2, 126.3 (2C), 119.5, 115.6 (2C), 70.2, 46.4, 41.5; IR *ν*_max_ (KBr): 3032, 1676, 1591, 1508, 1454, 1259, 1013, 883, 750, 694 cm^−1^; HRMS (ESITOF) *m*/*z* calcd for C_29_H_23_BrN_2_O_2_, [M + H]^+^ 511.1016, found 511.1021.
*3-Benzyl-2-(4-(benzyloxy)benzyl)-6-methoxyquinazolin-4(3H)-one* (**4r**). Oil, 37.9 mg, yield: 82%. ^1^H NMR (400 MHz, CDCl_3_) *δ* 7.72–7.64 (m, 2H), 7.44–7.25 (m, 9H), 7.13 (dd, *J* = 8.3, 3.0 Hz, 4H), 6.96–6.90 (m, 2H), 5.27 (s, 2H), 5.03 (s, 2H), 4.01 (s, 2H), 3.91 (s, 3H); ^13^C NMR (100 MHz, CDCl_3_) *δ* 162.7, 158.5, 158.2, 153.5, 142.1, 137.0, 136.4, 129.2 (2C), 129.1 (2C), 129.0, 128.7 (2C), 128.1, 127.8, 127.7, 127.6 (2C), 126.3 (2C), 124.9, 121.4, 115.5 (2C), 106.5, 70.2, 55.9, 46.4, 41.4; IR *ν*_max_ (KBr): 3032, 1667, 1591, 1489, 1360, 1240, 1026, 837, 750, 694 cm^−1^; HRMS (ESITOF) *m*/*z* calcd for C_30_H_26_N_2_O_3_, [M + H]^+^ 463.2016, found 463.2022.
*2-(4-(Benzyloxy)benzyl)-3-butylquinazolin-4(3H)-one* (**4s**). Oil, 39.0 mg, yield: 98%. ^1^H NMR (400 MHz, CDCl_3_) *δ* 8.26 (dd, *J* = 8.1, 2.9 Hz, 1H), 7.72 (tt, *J* = 8.3, 5.3 Hz, 2H), 7.49–7.43 (m, 1H), 7.43–7.28 (m, 5H), 7.18 (d, *J* = 7.7 Hz, 2H), 6.93 (dd, *J* = 7.9, 2.8 Hz, 2H), 5.04 (s, 2H), 4.18 (s, 2H), 4.01–3.88 (m, 2H), 1.60–1.49 (m, 2H), 1.36 (q, *J* = 7.5 Hz, 2H), 0.92 (td, *J* = 7.8, 2.5 Hz, 3H); ^13^C NMR (100 MHz, CDCl_3_) *δ* 162.5, 158.2, 155.6, 147.4, 137.0, 134.3, 129.4 (2C), 128.7 (2C), 128.1, 127.8, 127.6 (2C), 127.2, 126.9, 126.7, 120.9, 115.5 (2C), 70.2, 44.4, 41.8, 30.9, 20.4, 13.8; IR *ν*_max_ (KBr): 3034, 1672, 1589, 1510, 1474, 1259, 1175, 1022, 750, 696 cm^−1^; HRMS (ESITOF) *m*/*z* calcd for C_26H26_N_2_O_2_, [M + H]^+^ 399.2067, found 399.2065.
*3-Benzyl-2-(4-methoxybenzyl)quinazolin-4(3H)-one* (**4t**). Oil, 33.1 mg, yield: 93%. ^1^H NMR (400 MHz, CDCl_3_) *δ* 8.33 (dd, *J* = 8.1, 3.0 Hz, 1H), 7.75 (td, *J* = 9.6, 8.2, 3.9 Hz, 2H), 7.50 (t, *J* = 7.1 Hz, 1H), 7.37–7.23 (m, 3H), 7.16–7.12 (m, 4H), 6.86 (dt, *J* = 8.8, 2.1 Hz, 2H), 5.26 (s, 2H), 4.03 (s, 2H), 3.79 (s, 3H); ^13^C NMR (100 MHz, CDCl_3_) *δ* 162.9, 159.0, 155.9, 147.5, 136.3, 134.6, 129.2 (2C), 129.1 (2C), 127.7, 127.4, 127.3, 127.2, 127.0, 126.3 (2C), 120.7, 114.7 (2C), 55.4, 46.3, 41.6; IR *ν*_max_ (KBr): 3032, 1672, 1593, 1510, 1454, 1246, 1175, 1030, 750, 694 cm^−1^; HRMS (ESITOF) *m*/*z* calcd for C_23H20_N_2_O_2_, [M + H]^+^ 357.1598, found 357.1599.

#### Gram-Scale Synthesis and Synthesis of HBQ

*3-Benzyl-2-(4-(benzyloxy)benzyl)quinazolin-4(3H)-one* (**4j**). CuI (48 mg, 10 mol%) was added to an oven-dried 50 mL round-bottomed flask containing a mixture of 2-amino-*N*-benzylbenzamide **1a** (678 mg, 3.0 mmol, 1.0 equiv.), 1-(benzyloxy)-4-ethynylbenzene **2b** (686 mg, 3.3 mmol, 1.1 equiv.), TsN_3_ **3a** (650 mg, 3.3 mmol, 1.1 equiv.), and Et_3_N (333 mg, 3.3 mmol, 1.1 equiv.) in MeCN (20 mL). The reaction mixture was stirred for 12 h. After completion of the reaction as indicated by TLC, the solvent was removed by evaporation in a vacuum. The residue was directly purified by flash column chromatography on silica gel (eluting with hexanes/EtOAc = 2:1) to obtain **4j** (1.22 g, 94% yield) as oil.
*2-(4-Hydroxybenzyl)quinazolin-4(3H)-one (HBQ)*. To a stirred solution of **4j** (0.86 g, 2.0 mmol, 1.0 equiv.) in dry EtOAc (15 mL) was added palladium (10%) on carbon (15.0 mg). Then, the reaction mixture was stirred under an atmosphere of H_2_ at room temperature for 3 h. The reaction mixture was then filtered on a silica pad and rinsed with EtOAc. After evaporation of the solvent, the residue was purified by flash column chromatography on silica gel (eluting with petroleum ether/EtOAc = 1:1) to obtain **HBQ** as a white solid, 460 mg, yield: 92%, m.p: 210–212 °C (literature [15], m.p: no report). ^1^H NMR (400 MHz, CD_3_OD) *δ* 8.17 (dd, *J* = 8.2, 2.9 Hz, 1H), 7.84–7.76 (m, 1H), 7.71–7.65 (m, 1H), 7.50 (td, *J* = 7.8, 2.9 Hz, 1H), 7.22–7.15 (m, 2H), 6.75 (dt, *J* = 8.7, 2.1 Hz, 2H), 4.57 (s, 1H), 3.90 (s, 2H); ^13^C NMR (100 MHz, CD_3_OD) *δ* 164.4, 158.4, 157.8, 150.1, 136.0, 130.9 (2C), 127.8, 127.7, 127.6, 127.1, 121.8, 116.6 (2C), 41.5; IR *ν*_max_ (KBr): 3383, 2492, 1682, 1609, 1452, 1269, 1119, 972, 827, 756 cm^−1^; HRMS (ESITOF) *m*/*z* calcd for C_15H12_N_2_O_2_, [M + H]^+^ 253.0972, found 253.0969.

## 4. Conclusions

We have developed an oxidant-free and highly effective approach to synthesize phenolic quinazolin-4(3*H*)-ones via the CuAAC/ring cleavage reaction. *N*-sulfonylketenimine, generated by TsN_3_ and terminal alkynes, undergoes two nucleophilic additions by benzamides and anilines, and the sulfonyl group is eliminated through aromatization. More importantly, the protocol can be used to synthesize the natural product 2-(4-hydroxybenzyl)quinazolin-4(*3H*)-one and scaled up under mild conditions. Moreover, we expect that this methodology can be applied to building phenolic quinazolin-4(3*H*)-one block facility.

## Figures and Tables

**Table 1:** Optimization of catalytic conditions ^a^.

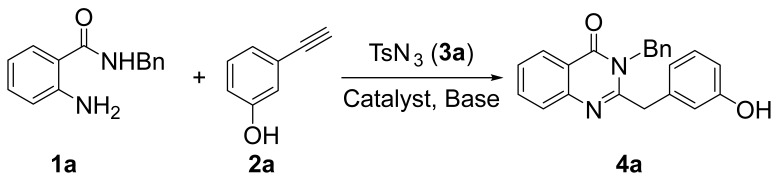				
**Entry**	**Cat.** **(10 mol%)**	**Base** **(0.11 mmol)**	**Solvent** **(2 mL)**	**Yield** **(%) ^b^**
1	CuI	Et_3_N	CHCl_3_	71
2	CuI	Et_3_N	DCE	74
3	CuI	Et_3_N	DCM	67
4	CuI	Et_3_N	Toluene	63
5	CuI	Et_3_N	MeCN	89
6	CuI	Et_3_N	THF	70
7	CuI	Et_3_N	DMSO	58
8	CuI	Et_3_N	DMF	63
9	CuI	Et_3_N	Dioxane	52
10	CuI	Et_3_N	EtOH	34
11	CuCl	Et_3_N	MeCN	85
12	CuBr	Et_3_N	MeCN	82
13	CuBr_2_	Et_3_N	MeCN	72
14	CuCl_2_·2H_2_O	Et_3_N	MeCN	68
15	Cu(OAc)_2_	Et_3_N	MeCN	74
16	Cu(acac)_2_	Et_3_N	MeCN	44
17	Cu(OTf)_2_	Et_3_N	MeCN	22
18	CuI	DAMP	MeCN	21
19	CuI	DIPEA	MeCN	86
20	CuI	NaOH	MeCN	12
21	CuI	Et_3_N	MeCN	89 ^c^

^a^ Reaction conditions: **1a** (0.10 mmol), **2a** (0.11 mmol), and the catalyst (10 mol%) and base (0.11 mmol) in the solvent (2 mL) were added with **3a** (0.11 mmol) and stirred at room temperature for 12 h. ^b^ Isolated yields. ^c^ MsN_3_ or PhSO_2_N_3_ was used instead of TsN_3_.

## Data Availability

Data are contained within the article and Supplementary Materials.

## Supplementary Materials

The following are available online at https://www.mdpi.com/article/10.3390/molecules28155734/s1. References [50,51] are cited in the supplementary materials.

## Sample Availability

Samples of the compounds **4a**–**4t** are available from the authors.
