# Supplementary material for: An Oxidant-Free and Mild Strategy for Quinazolin-4(3H)-One Synthesis via CuAAC/Ring Cleavage Reaction
Source: Molecules. 2023 Jul 28;28(15):5734. doi: 10.3390/molecules28155734 (PMC10420183; doi:10.3390/molecules28155734)

Supporting Information

An Oxidant-Free and Mild Strategy for Quinazolin-4(3*H*)-ones Synthesis via CuAAC/Ring Cleavage Reaction

Yueling He,#[a, b] Zhongtao Yang,#[a] Danyang Luo,[a] Xiai Luo,*[a, c] Xiaodong Chen*[d] and Weiguang Yang*[a]

[a] *The Marine Biomedical Research Institute of Guangdong Zhanjiang, Guangdong Medical University, Zhanjiang, 524023, China.*

[b] *School of Environmental science and Engineering, Donghua University, Shanghai, 201620, China.*

[c] *Hunan Province Key Laboratory for Synthetic Biology of Traditional Chinese Medicine, School of Pharmaceutical Sciences, Hunan University of Medicine, Huaihua, Hunan, China.*

[d] *Southern Marine Science and Engineering Guangdong Laboratory (Zhanjiang), Zhanjiang, Guangdong, 524023, China.*

# *These authors contributed equally*

E-mail: luoxiai83@163.com; chenxd@zjblab.com; 09ywg@163.com

Table of Contents

1. General Information ……………………………………………………….………...S2
2. General Procedure for the Synthesis of **4** …………………………………..…...S3
3. Characterization Data for Products **4**………………………………….…………...S4
4. Gram-scale Synthesis and Synthesis of HBQ………..…………………………S11
5. References…………………………………………………………….……...S12
6. Copies of NMR Spectra……………………………………………….……...S13

1. General Information

^{1}H NMR spectrum were recorded on a Bruker DPX 400 MHz spectrometer in $CDCl_3$. Chemical shifts were reported in ppm with the internal TMS signal at 0.0 ppm as a standard. The spectra are interpreted as: s, singlet; bs, broad singlet; d, doublet; t, triplet; q, quartet; m, multiplet; dd, double doublet; ddd, double double doublet; dt, double triplet; ddt, double double triplet; tt, triple triplet, td, triple doublet; coupling constant(s) *J* are reported in Hz and relative integrations are reported. ^{13}C NMR (100 MHz) spectrum were recorded on a Bruker DPX 400 MHz spectrometer in $CDCl_3$. Chemical shifts were reported in ppm with the internal chloroform signal at 77.16 ppm as a standard; additionally, HBQ using CD_3OD residual nondeuterated solvent as internal standard CD_3OD: δ 3.31 for ^{1}H and 49.00 ppm for ^{13}C. Melting points were obtained in open capillary tubes using SGW X-4 micro melting point apparatus which were uncorrected. IR spectra were obtained with Bruker Tensor-27 FT-IR Spectrometer. Mass spectrum were recorded on TOF mass spectrometer. The starting materials 2-amino-*N*-benzylbenzamide derivatives **1** were all known and prepared according to the literature procedures [1]. Terminal alkynes **2**, TsN_3 **3a**, and other reagents were purchased from Adamas-beta and other suppliers and used without further purification.

2. General Procedure for the Synthesis of 4

Copper catalyzed one pot synthesis of quinazoline 4-(3*H*)-ones 4:

To a solution of 2-amino-*N*-benzylbenzamides **1** (0.1 mmol, 1.0 equiv.), phenyl acetylenes **2** (0.11 mmol, 1.1 equiv.), TsN_3 **3a** (21.7 mg, 0.11 mmol, 1.1 equiv.) and Et_3N (11.1 mg, 0.11 mmol, 1.1 equiv.) in MeCN (2 mL) was added CuI (48 mg, 10 mol%). The reaction mixture was stirred at room temperature for 12 hours. After completion of the reaction as indicated by TLC, the solvent was removed by evaporating in vacuum. The residue was directly purified by flash column chromatography on silica gel (eluting with hexanes/EtOAc = 2:1) to afford the corresponding product **4**.

3. Characterization Data for Products 4

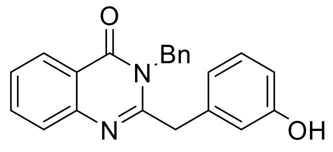

4a

3-Benzyl-2-(3-hydroxybenzyl)quinazolin-4(3*H*)-one (4a)

white solid, 30.5 mg, yield: 89%, m.p: 180–182 °C. **^{1}H NMR** (400 MHz, $CDCl_3$) δ 9.86 (s, 1H), 8.08–7.93 (m, 1H), 7.59–7.52 (m, 1H), 7.42 (dt, J = 9.6, 4.7 Hz, 2H), 7.36–7.20 (m, 4H), 7.10 (d, J = 7.2 Hz, 2H), 6.82 (d, J = 5.4 Hz, 2H), 6.74 (d, J = 7.7 Hz, 1H), 5.13 (s, 2H), 4.00 (s, 2H); **^{13}C NMR** (100 MHz, $CDCl_3$) δ 161.8, 158.3, 156.8, 145.2, 135.8 (2C), 134.9, 131.0, 129.2 (2C), 128.0, 127.9, 127.5, 126.4 (2C), 124.9, 119.8, 119.7, 115.4, 113.6, 46.3, 41.5; **IR** ν_{max} (KBr): 3308, 2928, 1682, 1591, 1456, 1265, 1165, 976, 775, 731 cm^{-1}; **HRMS** (ESITOF) m/z calcd for $C_{22}H_{18}N_2O_2$, $[M+H]^+$ 343.1441, found 1443.

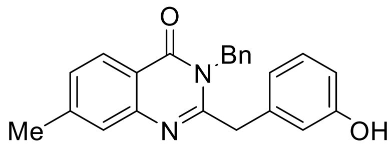

4b

3-Benzyl-2-(3-hydroxybenzyl)-7-methylquinazolin-4(3*H*)-one (4b)

white solid, 30.6 mg, yield: 86%, m.p: 184–186 °C. **^{1}H NMR** (400 MHz, $CDCl_3$) δ 9.86 (s, 1H), 7.90 (dd, J = 8.5, 2.9 Hz, 1H), 7.38–7.28 (m, 3H), 7.27–7.20 (m, 2H), 7.15 (d, J = 2.5 Hz, 1H), 7.13–7.08 (m, 2H), 6.80 (d, J = 4.9 Hz, 2H), 6.73 (d, J = 7.6 Hz, 1H), 5.12 (s, 2H), 3.97 (s, 2H), 2.37 (s, 3H); **^{13}C NMR** (100 MHz, $CDCl_3$) δ 161.7, 158.4, 156.8, 146.4, 145.4, 136.0, 135.9, 130.9, 129.9, 129.2, 129.0, 128.0, 127.8, 126.6, 126.4, 124.6, 119.7, 117.3, 115.3, 113.6, 46.0, 41.4, 22.1; **IR** ν_{max} (KBr): 3055, 1682, 1592, 1456, 1342, 1265, 1163, 974, 879, 737 cm^{-1}; **HRMS** (ESITOF) m/z calcd for $C_{23}H_{20}N_2O_2$, $[M+H]^+$ 357.1598, found 357.1599.

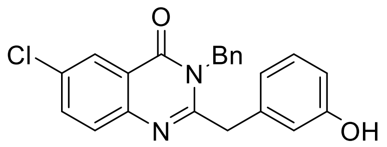

4c

3-Benzyl-6-chloro-2-(3-hydroxybenzyl)quinazolin-4(3*H*)-one (4c)

white solid, 34.2 mg, yield: 91%, m.p: 197–199 °C. **^{1}H NMR** (400 MHz, $CDCl_3$) δ 9.23 (s, 1H), 8.11 (d, J = 2.6 Hz, 1H), 7.50 (dt, J = 8.3, 2.5 Hz, 1H), 7.37–7.21 (m, 5H), 7.10 (dd, J = 7.7, 3.2 Hz, 2H), 6.81 (d, J = 8.5 Hz, 1H), 6.78–6.72 (m, 2H), 5.15 (s, 2H), 4.00 (s, 2H); **^{13}C NMR** (100 MHz, $CDCl_3$) δ 160.9, 158.1, 157.2, 143.9, 135.7, 135.5, 135.4, 133.5, 131.1, 129.3 (2C), 128.2, 127.0, 126.7, 126.5 (2C), 120.9, 120.0, 115.5, 113.5, 46.5, 41.5; **IR** ν_{max} (KBr): 3034, 2947, 1688, 1587, 1473, 1277, 1155, 980, 764, 717 cm^{-1}; **HRMS** (ESITOF) m/z calcd for $C_{22}H_{17}ClN_2O_2$, $[M+H]^+$ 377.1051, found 377.1056.

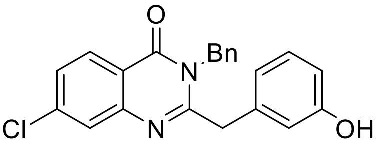

4d

3-Benzyl-7-chloro-2-(3-hydroxybenzyl)quinazolin-4(3*H*)-one (4d)

white solid, 32.7 mg, yield: 87%, m.p: 190–192 °C. **^{1}H NMR** (400 MHz, $CDCl_3$) δ 8.23 (s, 1H), 8.14–8.03 (m, 1H), 7.47 (d, J = 2.7 Hz, 1H), 7.44–7.38 (m, 1H), 7.37–7.29 (m, 3H), 7.28–7.22 (m, 1H), 7.13 (d, J = 7.1 Hz, 2H), 6.81 (d, J = 8.3 Hz, 1H), 6.75 (d, J = 15.3 Hz, 2H), 5.17 (s, 2H), 4.00 (s, 2H); **^{13}C NMR** (100 MHz, $CDCl_3$) δ 161.3, 156.0, 157.7, 146.8, 141.3, 135.8, 135.7, 131.0, 129.3 (2C), 129.2, 128.1 (2C), 126.4 (2C), 125.3, 120.2, 118.5, 115.4, 113.9, 46.5, 41.7; **IR** ν_{max} (KBr): 2924, 1684, 1601, 1456, 1331, 1232, 1265, 1159, 974, 731 cm^{-1}; **HRMS** (ESITOF) m/z calcd for $C_{22}H_{17}ClN_2O_2$, $[M+H]^+$ 377.1051, found 377.1056.

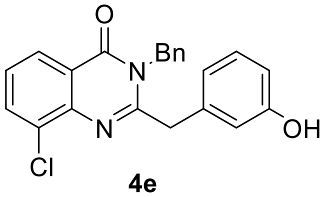

4e

3-Benzyl-8-chloro-2-(3-hydroxybenzyl)quinazolin-4(3*H*)-one (4e)

white solid, 30.1 mg, yield: 80%, m.p: 197–199 °C. **^{1}H NMR** (400 MHz, $CDCl_3$) δ 8.21 (dd, J = 8.1, 3.0 Hz, 1H), 7.75 (dd, J = 7.9, 3.0 Hz, 1H), 7.42–7.27 (m, 4H), 7.18 (td, J = 7.7, 7.2, 4.2 Hz, 1H), 7.13 (d, J = 7.5 Hz, 2H), 6.83 (s, 1H), 6.75 (t, J = 9.9 Hz, 2H), 5.98 (s, 1H), 5.25 (s, 2H), 4.09 (s, 2H); **^{13}C NMR** (100 MHz, $CDCl_3$) δ 162.2, 156.9, 156.7, 143.9, 136.5, 135.8, 134.9, 131.4, 130.5, 129.2 (2C), 128.0, 127.1, 126.3 (2C), 126.2, 122.2, 120.5, 115.1, 114.8, 46.6, 41.9; **IR** ν_{max} (KBr): 3007, 1676, 1580, 1445, 1389, 1275, 1159, 980, 849, 764 cm^{-1}; **HRMS** (ESITOF) m/z calcd for $C_{22}H_{17}ClN_2O_2$, $[M+H]^+$ 377.1051, found 377.1056.

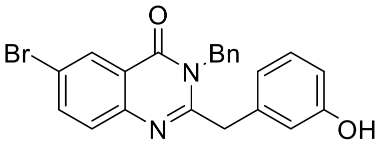

4f

3-Benzyl-6-bromo-2-(3-hydroxybenzyl)quinazolin-4(3*H*)-one (4f)

white solid, 38.6 mg, yield: 92%, m.p: 176–178 °C. **^{1}H NMR** (400 MHz, $CDCl_3$) δ 9.33 (s, 1H), 8.30 (q, J = 2.2 Hz, 1H), 7.68–7.57 (m, 1H), 7.38–7.21 (m, 5H), 7.10 (d, J = 7.2 Hz, 2H), 6.81 (d, J = 8.4 Hz, 1H), 6.75 (d, J = 10.2 Hz, 2H), 5.15 (s, 2H), 4.00 (s, 2H); **^{13}C NMR** (100 MHz, $CDCl_3$) δ 160.7, 158.2, 157.4, 144.2, 138.2, 135.6, 135.5, 131.1, 130.3, 129.3 (2C), 128.2, 126.7, 126.5 (2C), 121.3, 121.2, 120.0, 115.5, 113.4, 46.5, 41.5; **IR** ν_{max} (KBr): 3026, 1684, 1587, 1456, 1389, 1277, 1153, 966, 831, 750 cm^{-1}; **HRMS** (ESITOF) m/z calcd for $C_{22}H_{17}BrN_2O_2$, $[M+H]^+$ 421.0546, found 421.0551.

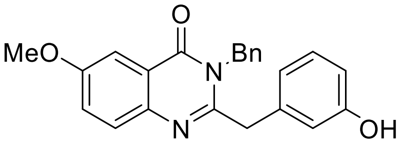

4g

3-Benzyl-2-(3-hydroxybenzyl)-6-methoxyquinazolin-4(3*H*)-one (4g)

white solid, 28.3 mg, yield: 76%, m.p: 183–185 °C. **^{1}H NMR** (400 MHz, $CDCl_3$) δ 10.06 (s, 1H), 7.42 (q, J = 2.7 Hz, 1H), 7.37–7.19 (m, 5H), 7.11 (td, J = 5.6, 2.7 Hz, 3H), 6.81 (d, J = 2.8 Hz, 2H), 6.73 (d, J = 7.6 Hz, 1H), 5.13 (s, 2H), 3.99 (s, 2H), 3.97 (s, 3H); **^{13}C NMR** (100 MHz, $CDCl_3$) δ 161.7, 158.5, 158.4, 154.4, 139.6, 136.1, 136.0, 130.9, 129.8, 129.2, 128.0, 126.6, 126.4 (2C), 124.1, 120.8, 119.7, 115.4, 113.6, 108.1, 55.8, 46.4, 41.2; **IR** ν_{max} (KBr): 3005, 1670, 1593, 1495, 1456, 1362, 1275, 1155, 1028, 750 cm^{-1}; **HRMS** (ESITOF) m/z calcd for $C_{23}H_{20}N_2O_3$, $[M+H]^+$ 373.1547, found 373.1549.

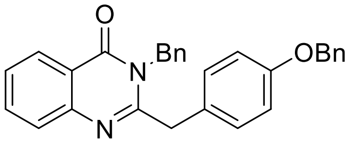

4j

3-Benzyl-2-(4-(benzyloxy)benzyl)quinazolin-4(3*H*)-one (4j)

oil, 40.6 mg, yield: 94%. **^{1}H NMR** (400 MHz, $CDCl_3$) δ 8.33 (dd, J = 8.1, 2.8 Hz, 1H), 7.82–7.70 (m, 2H), 7.50 (d, J = 8.1 Hz, 1H), 7.44–7.21 (m, 8H), 7.17–7.11 (m, 4H), 6.93 (dd, J = 7.8, 3.1 Hz, 2H), 5.26 (s, 2H), 5.04 (s, 2H), 4.02 (s, 2H); **^{13}C NMR** (100 MHz, $CDCl_3$) δ 162.8, 158.2, 155.9, 147.4, 136.9, 136.3, 134.6, 129.3 (2C), 129.1 (2C), 128.7 (2C), 128.1, 127.7, 127.6 (2C), 127.5, 127.4, 127.3, 127.0, 126.3 (2C), 120.6, 115.6 (2C), 70.2, 46.3, 41.6; **IR** ν_{max} (KBr): 3032, 1672, 1591, 1508, 1454, 1240, 1172, 1013, 750 694 cm^{-1}; **HRMS** (ESITOF) m/z calcd for $C_{29}H_{24}N_2O_2$, $[M+H]^+$ 433.1911, found 433.1910.

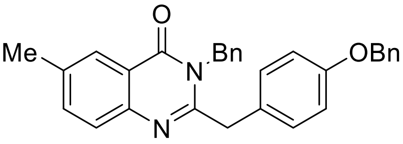

4k

3-Benzyl-2-(4-(benzyloxy)benzyl)-6-methylquinazolin-4(3*H*)-one (4k)

white solid, 41.1 mg, yield: 92%, m.p: 137–139 °C. **^{1}H NMR** (400 MHz, $CDCl_3$) δ 8.12 (s, 1H), 7.64 (dd, J = 8.3, 2.8 Hz, 1H), 7.61–7.57 (m, 1H), 7.45–7.22 (m, 8H), 7.13 (d, J = 7.3 Hz, 4H), 6.92 (dd, J = 8.3, 3.0 Hz, 2H), 5.26 (s, 2H), 5.04 (s, 2H), 4.01 (s, 2H), 2.50 (s, 3H); **^{13}C NMR** (100 MHz, $CDCl_3$) δ 162.9, 158.2, 155.0, 145.5, 137.2, 137.0, 136.4, 136.0, 129.2 (2C), 129.1 (2C), 128.7 (2C), 128.1, 127.7 (2C), 127.6 (2C), 127.2, 126.7, 126.3 (2C), 120.4, 115.6 (2C), 70.2, 46.2, 41.5, 21.5; **IR** ν_{max} (KBr): 3032, 1670, 1591, 1508, 1454, 1340, 1275, 1013, 831, 750 cm^{-1}; **HRMS** (ESITOF) m/z calcd for $C_{30}H_{26}N_2O_2$, $[M+H]^+$ 447.2067, found 447.2069.

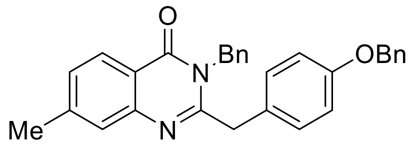

4l

3-Benzyl-2-(4-(benzyloxy)benzyl)-7-methylquinazolin-4(3*H*)-one (4l)

oil, 40.2 mg, yield: 90%. **^{1}H NMR** (400 MHz, $CDCl_3$) δ 8.21 (dd, J = 8.5, 3.0 Hz, 1H), 7.54 (s, 1H), 7.44–7.23 (m, 9H), 7.13 (d, J = 7.2 Hz, 4H), 6.95–6.89 (m, 2H), 5.25 (s, 2H), 5.03 (s, 2H), 4.00 (s, 2H), 2.51 (s, 3H); **^{13}C NMR** (100 MHz, $CDCl_3$) δ 162.8, 158.2, 155.9, 147.5, 145.6, 137.0, 136.5, 129.3 (2C), 129.1 (2C), 128.7 (2C), 128.5, 128.1, 127.7, 127.6, 127.5 (2C), 127.1, 127.0, 126.3 (2C), 118.2, 115.6 (2C), 70.2, 46.1, 41.6, 22.0; **IR** ν_{max} (KBr): 3032, 1672, 1593, 1508, 1454, 1259, 1173, 1011, 750, 696 cm^{-1}; **HRMS** (ESITOF) m/z calcd for $C_{30}H_{26}N_2O_2$, $[M+H]^+$ 447.2067, found 447.2069.

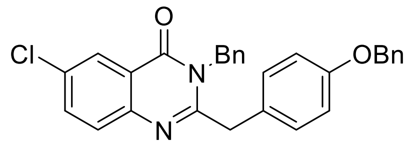

4m

3-Benzyl-2-(4-(benzyloxy)benzyl)-6-chloroquinazolin-4(3*H*)-one (4m)

white solid, 44.7 mg, yield: 96%, m.p: 174–176 °C. **^{1}H NMR** (400 MHz, $CDCl_3$) δ 8.20 (s, 1H), 7.66–7.55 (m, 2H), 7.38–7.15 (m, 8H), 7.04 (dd, J = 8.1, 2.9 Hz, 4H), 6.85 (dd, J = 8.7, 3.0 Hz, 2H), 5.17 (s, 2H), 4.96 (s, 2H), 3.93 (s, 2H); **^{13}C NMR** (100 MHz, $CDCl_3$) δ 161.9, 158.3, 156.2, 146.0, 136.9, 136.0, 135.0, 132.7, 129.3 (2C), 129.2 (2C), 129.1, 128.7 (2C), 128.2, 127.9, 127.6 (2C), 127.2, 126.6, 126.3 (2C), 121.7, 115.6 (2C), 70.2, 46.4, 41.5; **IR** ν_{max} (KBr): 3034, 1676, 1591, 1508, 1472, 1335, 1275, 1013, 835, 750 cm^{-1}; **HRMS** (ESITOF) m/z calcd for $C_{29}H_{23}ClN_2O_2$, $[M+H]^+$ 467.1521, found 467.1528.

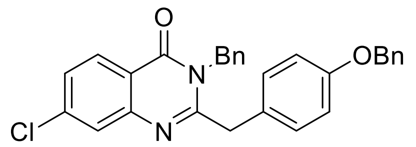

4n

3-Benzyl-2-(4-(benzyloxy)benzyl)-7-chloroquinazolin-4(3*H*)-one (4n)

oil, 43.4 mg, yield: 93%. **^{1}H NMR** (400 MHz, $CDCl_3$) δ 8.24 (dd, J = 9.0, 3.1 Hz, 1H), 7.73 (d, J = 3.0 Hz, 1H), 7.47–7.23 (m, 9H), 7.13 (d, J = 6.8 Hz, 4H), 6.93 (dd, J = 8.1, 3.2 Hz, 2H), 5.25 (s, 2H), 5.04 (s, 2H), 4.00 (s, 2H); **^{13}C NMR** (100 MHz, $CDCl_3$) δ 162.3, 158.3, 157.3, 148.4, 140.8, 136.9, 136.1, 129.3 (2C), 129.2 (2C), 128.8, 128.7 (2C), 128.2, 127.9, 127.6 (2C), 127.5, 127.2, 127.0, 126.3 (2C), 119.1, 115.6 (2C), 70.2, 46.3, 41.5; **IR** ν_{max} (KBr): 3034, 1676, 1591, 1508, 1454, 1383, 1240, 1013, 748, 694 cm^{-1}; **HRMS** (ESITOF) m/z calcd for $C_{29}H_{23}ClN_2O_2$, $[M+H]^+$ 467.1521, found 467.1528.

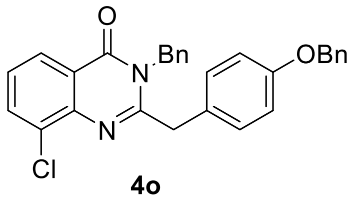

4o

3-Benzyl-2-(4-(benzyloxy)benzyl)-8-chloroquinazolin-4(3*H*)-one (4o)

white solid, 38.2 mg, yield: 82%, m.p: 111–113 °C. **^{1}H NMR** (400 MHz, $CDCl_3$) δ 8.22 (dd, J = 8.0, 2.8 Hz, 1H), 7.84 (dd, J = 7.9, 2.8 Hz, 1H), 7.44–7.35 (m, 5H), 7.34–7.26 (m, 4H), 7.16 (d, J = 7.8 Hz, 2H), 7.12 (d, J = 6.7 Hz, 2H), 6.93 (dd, J = 8.0, 2.6 Hz, 2H), 5.26 (s, 2H), 5.04 (s, 2H), 4.07 (s, 2H); **^{13}C NMR** (100 MHz, $CDCl_3$) δ 162.3, 158.3, 156.8, 144.2, 137.0, 135.9, 134.8, 129.5 (2C), 129.2 (2C), 128.91, 128.7 (2C), 128.1, 127.9, 127.6 (2C), 127.3, 126.9, 126.3 (2C), 126.1, 122.2, 115.6 (2C), 70.2, 46.5, 41.7; **IR** ν_{max} (KBr): 3030, 1676, 1591, 1508, 1445, 1261, 1163, 987, 748, 696 cm^{-1}; **HRMS** (ESITOF) m/z calcd for $C_{29}H_{23}ClN_2O_2$, $[M+H]^+$ 467.1521, found 467.1528.

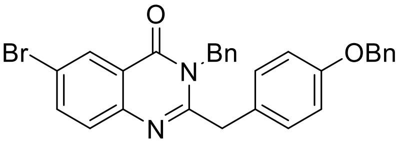

4p

3-Benzyl-2-(4-(benzyloxy)benzyl)-6-bromoquinazolin-4(3*H*)-one (4p)

white solid, 49.5 mg, yield: 97%, m.p: 167–169 °C. **^{1}H NMR** (400 MHz, $CDCl_3$) δ 8.45 (t, J = 2.4 Hz, 1H), 7.83 (dd, J = 8.7, 2.6 Hz, 1H), 7.59 (dd, J = 9.4, 3.0 Hz, 1H), 7.44–7.29 (m, 8H), 7.12 (d, J = 6.8 Hz, 4H), 6.93 (dd, J = 7.8, 2.8 Hz, 2H), 5.25 (s, 2H), 5.04 (s, 2H), 4.00 (s, 2H); **^{13}C NMR** (100 MHz, $CDCl_3$) δ 161.7, 158.3, 156.3, 146.3, 137.7, 136.9, 136.0, 129.8, 129.3 (2C), 129.2, 129.1 (2C), 128.7 (2C), 128.2, 127.9, 127.6 (2C), 127.2, 126.3 (2C), 122.0, 120.4, 115.6 (2C), 70.2, 46.4, 41.5; **IR** ν_{max} (KBr): 2905, 1676, 1589, 1510, 1467, 1333, 1275, 985, 750, 692 cm^{-1}; **HRMS** (ESITOF) m/z calcd for $C_{29}H_{23}BrN_2O_2$, $[M+H]^+$ 511.1016, found 511.1021.

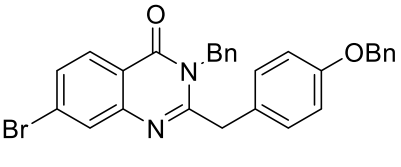

4q

3-Benzyl-2-(4-(benzyloxy)benzyl)-7-bromoquinazolin-4(3*H*)-one (4q)

oil, 45.4 mg, yield: 89%. **^{1}H NMR** (400 MHz, $CDCl_3$) δ 8.16 (dd, J = 8.8, 3.0 Hz, 1H), 7.91 (s, 1H), 7.58 (dd, J = 8.6, 2.7 Hz, 1H), 7.45–7.23 (m, 8H), 7.13 (d, J = 6.9 Hz, 4H), 6.97–6.90 (m, 2H), 5.25 (s, 2H), 5.04 (s, 2H), 4.00 (s, 2H); **^{13}C NMR** (100 MHz, $CDCl_3$) δ 162.4, 158.3, 157.3, 148.4, 136.9, 136.0, 130.3, 130.2, 129.4 (2C), 129.3, 129.2 (2C), 128.8, 128.7 (2C), 128.2, 127.9, 127.6 (2C), 127.2, 126.3 (2C), 119.5, 115.6 (2C), 70.2, 46.4, 41.5; **IR** ν_{max} (KBr): 3032, 1676, 1591, 1508, 1454, 1259, 1013, 883, 750, 694 cm^{-1}; **HRMS** (ESITOF) m/z calcd for $C_{29}H_{23}BrN_2O_2$, $[M+H]^+$ 511.1016, found 511.1021.

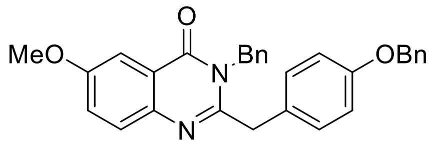

4r

3-Benzyl-2-(4-(benzyloxy)benzyl)-6-methoxyquinazolin-4(3*H*)-one (4r)

oil, 37.9 mg, yield: 82%. **^{1}H NMR** (400 MHz, $CDCl_3$) δ 7.72–7.64 (m, 2H), 7.44–7.25 (m, 9H), 7.13 (dd, J = 8.3, 3.0 Hz, 4H), 6.96–6.90 (m, 2H), 5.27 (s, 2H), 5.03 (s, 2H), 4.01 (s, 2H), 3.91 (s, 3H); **^{13}C NMR** (100 MHz, $CDCl_3$) δ 162.7, 158.5, 158.2, 153.5, 142.1, 137.0, 136.4, 129.2 (2C), 129.1 (2C), 129.0, 128.7 (2C), 128.1, 127.8, 127.7, 127.6 (2C), 126.3 (2C), 124.9, 121.4, 115.5 (2C), 106.5, 70.2, 55.9, 46.4, 41.4; **IR** ν_{max} (KBr): 3032, 1667, 1591, 1489, 1360, 1240, 1026, 837, 750, 694 cm^{-1}; **HRMS** (ESITOF) m/z calcd for $C_{30}H_{26}N_2O_3$, $[M+H]^+$ 463.2016, found 463.2022.

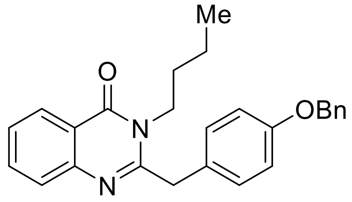

4s

2-(4-(Benzyloxy)benzyl)-3-butylquinazolin-4(3*H*)-one (4s)

oil, 39.0 mg, yield: 98%. **^{1}H NMR** (400 MHz, $CDCl_3$) δ 8.26 (dd, J = 8.1, 2.9 Hz, 1H), 7.72 (tt, J = 8.3, 5.3 Hz, 2H), 7.49–7.43 (m, 1H), 7.43–7.28 (m, 5H), 7.18 (d, J = 7.7 Hz, 2H), 6.93 (dd, J = 7.9, 2.8 Hz, 2H), 5.04 (s, 2H), 4.18 (s, 2H), 4.01–3.88 (m, 2H), 1.60–1.49 (m, 2H), 1.36 (q, J = 7.5 Hz, 2H), 0.92 (td, J = 7.8, 2.5 Hz, 3H); **^{13}C NMR** (100 MHz, $CDCl_3$) δ 162.5, 158.2, 155.6, 147.4, 137.0, 134.3, 129.4 (2C), 128.7 (2C), 128.1, 127.8, 127.6 (2C), 127.2, 126.9, 126.7, 120.9, 115.5 (2C), 70.2, 44.4, 41.8, 30.9, 20.4, 13.8; **IR** ν_{max} (KBr): 3034, 1672, 1589, 1510, 1474, 1259, 1175, 1022, 750, 696 cm^{-1}; **HRMS** (ESITOF) m/z calcd for $C_{26}H_{26}N_2O_2$, $[M+H]^+$ 399.2067, found 399.2065.

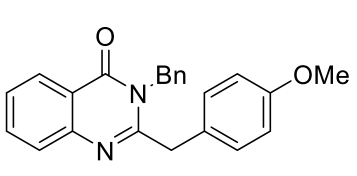

4t

3-Benzyl-2-(4-methoxybenzyl)quinazolin-4(3*H*)-one (4t)

oil, 33.1 mg, yield: 93%. **^{1}H NMR** (400 MHz, $CDCl_3$) δ 8.33 (dd, J = 8.1, 3.0 Hz, 1H), 7.75 (td, J = 9.6, 8.2, 3.9 Hz, 2H), 7.50 (t, J = 7.1 Hz, 1H), 7.37–7.23 (m, 3H), 7.16–7.12 (m, 4H), 6.86 (dt, J = 8.8, 2.1 Hz, 2H), 5.26 (s, 2H), 4.03 (s, 2H), 3.79 (s, 3H); **^{13}C NMR** (100 MHz, $CDCl_3$) δ 162.9, 159.0, 155.9, 147.5, 136.3, 134.6, 129.2 (2C), 129.1 (2C), 127.7, 127.4, 127.3, 127.2, 127.0, 126.3 (2C), 120.7, 114.7 (2C), 55.4, 46.3, 41.6; **IR** ν_{max} (KBr): 3032, 1672, 1593,

1510, 1454, 1246, 1175, 1030, 750, 694 cm^{-1}; **HRMS** (ESITOF) m/z calcd for $C_{23}H_{20}N_2O_2$, $[M+H]^+$ 357.1598, found 357.1599.

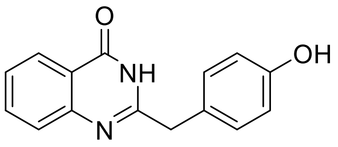

HBQ

2-(4-Hydroxybenzyl)quinazolin-4(3*H*)-one (HBQ)

white solid, 23.2 mg, yield: 92%, m.p: 210–212 °C (literature [15], m.p: no report). **^{1}H NMR** (400 MHz, CD_3OD) δ 8.17 (dd, J = 8.2, 2.9 Hz, 1H), 7.84–7.76 (m, 1H), 7.71–7.65 (m, 1H), 7.50 (td, J = 7.8, 2.9 Hz, 1H), 7.22–7.15 (m, 2H), 6.75 (dt, J = 8.7, 2.1 Hz, 2H), 4.57 (s, 1H), 3.90 (s, 2H); **^{13}C NMR** (100 MHz, CD_3OD) δ 164.4, 158.4, 157.8, 150.1, 136.0, 130.9 (2C), 127.8, 127.7, 127.6, 127.1, 121.8, 116.6 (2C), 41.5; **IR** ν_{max} (KBr): 3383, 2492, 1682, 1609, 1452, 1269, 1119, 972, 827, 756 cm^{-1}; **HRMS** (ESITOF) m/z calcd for $C_{15}H_{12}N_2O_2$, $[M+H]^+$ 253.0972, found 253.0969.

4. Gram-scale Synthesis and Synthesis of HBQ

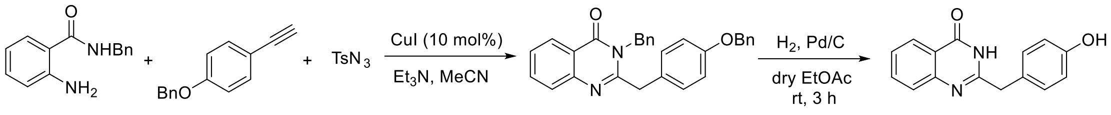

3-Benzyl-2-(4-(benzyloxy)benzyl)quinazolin-4(3*H*)-one (4j)

At room temperature, to an oven-dried 50 mL round bottomed flask equipped with a mixture of the 2-amino-*N*-benzylbenzamide **1a** (678 mg, 3.0 mmol, 1.0 equiv.), 1-(benzyloxy)-4-ethynylbenzene **2b** (686 mg, 3.3 mmol, 1.1 equiv.), TsN_3 **3a** (650 mg, 3.3 mmol, 1.1 equiv.) and Et_3N (333 mg, 3.3 mmol, 1.1 equiv.) in MeCN (20 mL) was added CuI (48 mg, 10 mol%). The reaction mixture was stirred for 12 hours. After completion of the reaction as indicated by TLC, the solvent was removed by evaporating in vacuum. The residue was directly purified by flash column chromatography on silica gel (eluting with hexanes/EtOAc = 2:1) to give **4ab** (1.22 g, 94% yield) as oil.

2-(4-Hydroxybenzyl)quinazolin-4(3*H*)-one (HBQ)

To a stirred solution of **4j** (0.86 g, 2.0 mmol, 1.0 equiv.) in dry EtOAc (15 mL) was added palladium (10%) on carbon (15.0 mg). Then the reaction mixture was stirred under an atmosphere of H_2 at room temperature for 3 h. The reaction mixture was then filtered on a silica pad and rinsed with EtOAc. After evaporation of solvent, the residue was was purified by flash column chromatography on silica gel (eluting with petroleum ether/EtOAc = 1:1) to give **HBQ** (0.46 g, 92% yield) as a white solid.

white solid, 460 mg, yield: 92%, m.p: 210–212 °C. **^{1}H NMR** (400 MHz, CD_3OD) δ 8.17 (dd, J = 8.2, 2.9 Hz, 1H), 7.84–7.76 (m, 1H), 7.71–7.65 (m, 1H), 7.50 (td, J = 7.8, 2.9 Hz, 1H), 7.22–7.15 (m, 2H), 6.75 (dt, J = 8.7, 2.1 Hz, 2H), 4.57 (s, 1H), 3.90 (s, 2H); **^{13}C NMR** (100 MHz, CD_3OD) δ 164.4, 158.4, 157.8, 150.1, 136.0, 130.9 (2C), 127.8, 127.7, 127.6, 127.1, 121.8, 116.6 (2C), 41.5; **IR** ν_{max} (KBr): 3383, 2492, 1682, 1609, 1452, 1269, 1119, 972, 827, 756 cm^{-1}; **HRMS** (ESITOF) m/z calcd for $C_{15}H_{12}N_2O_2$, $[M+H]^+$ 253.0972, found 253.0969.

5. References

[1] (a) Y. Bao, Y. Yan, K. Xu, J. Su, Z. Zha, Z. Wang. Copper-Catalyzed Radical Methylation/C–H Amination/Oxidation Cascade for the Synthesis of Quinazolinones. *J. Org. Chem*. **2015**, *80*, 4736-4742; (b) S. M. Patel, H. Chada, S. Biswal, S. Sharma, D. S. Sharada. Copper-Catalyzed Intramolecular α-C–H Amination via Ring Opening Cyclization Strategy to Quinazolin-4-ones: Development and Application in Rutaecarpine Synthesis. *Synthesis* **2019**, *51*, 3160-3170.

6. Copies of NMR spectra

^{1}H NMR spectrum of compound **4a** ($CDCl_3$)

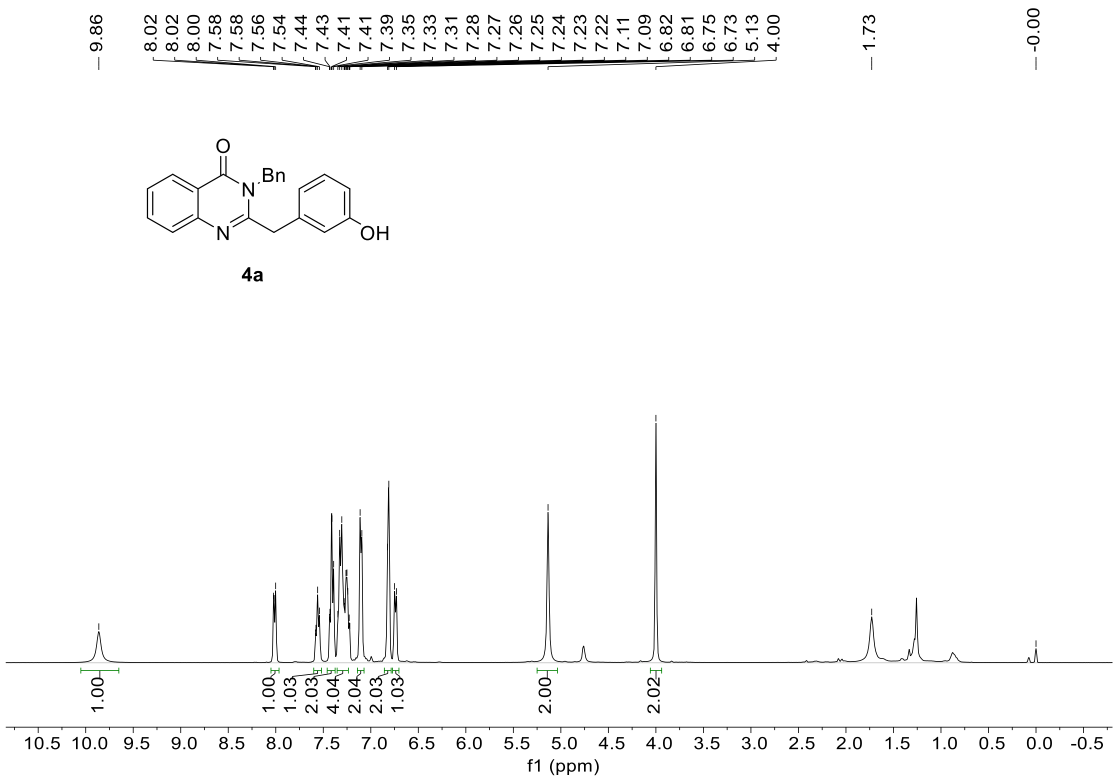

^{13}C NMR spectrum of compound **4a** ($CDCl_3$)

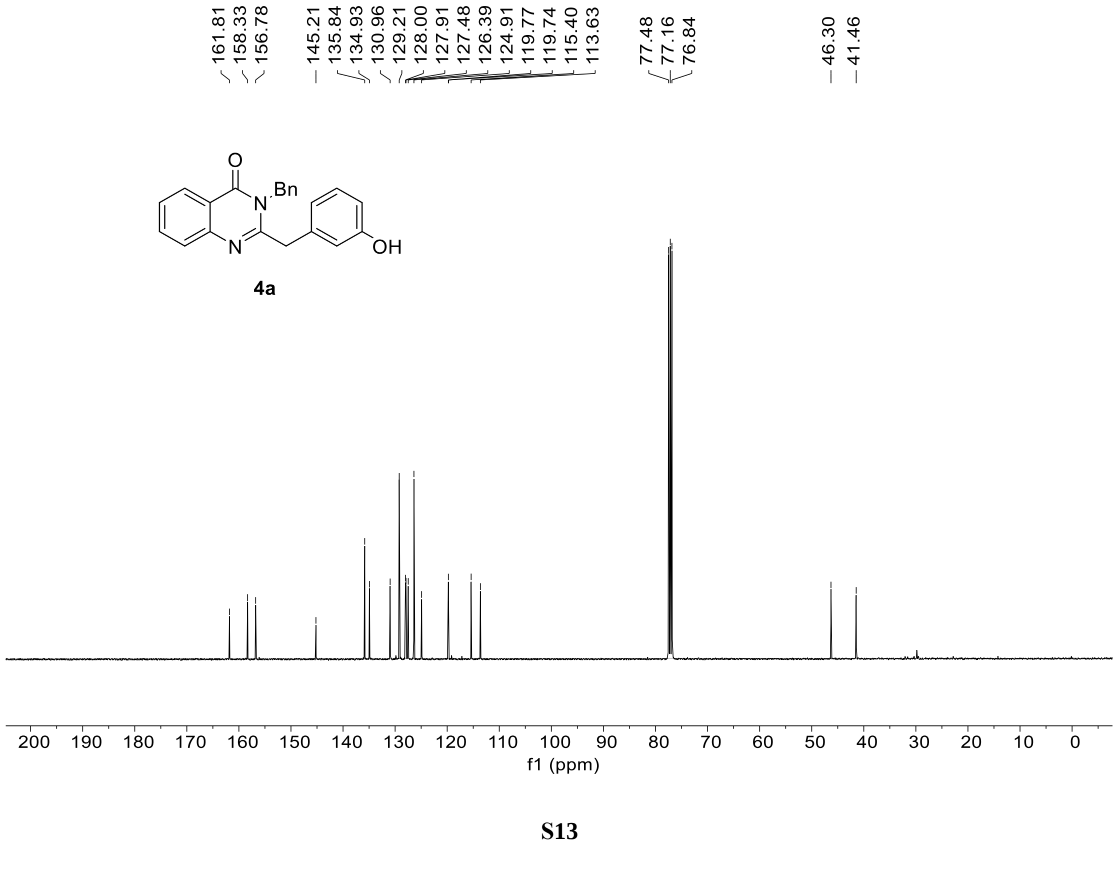

^{1}H NMR spectrum of compound **4b** ($CDCl_3$)

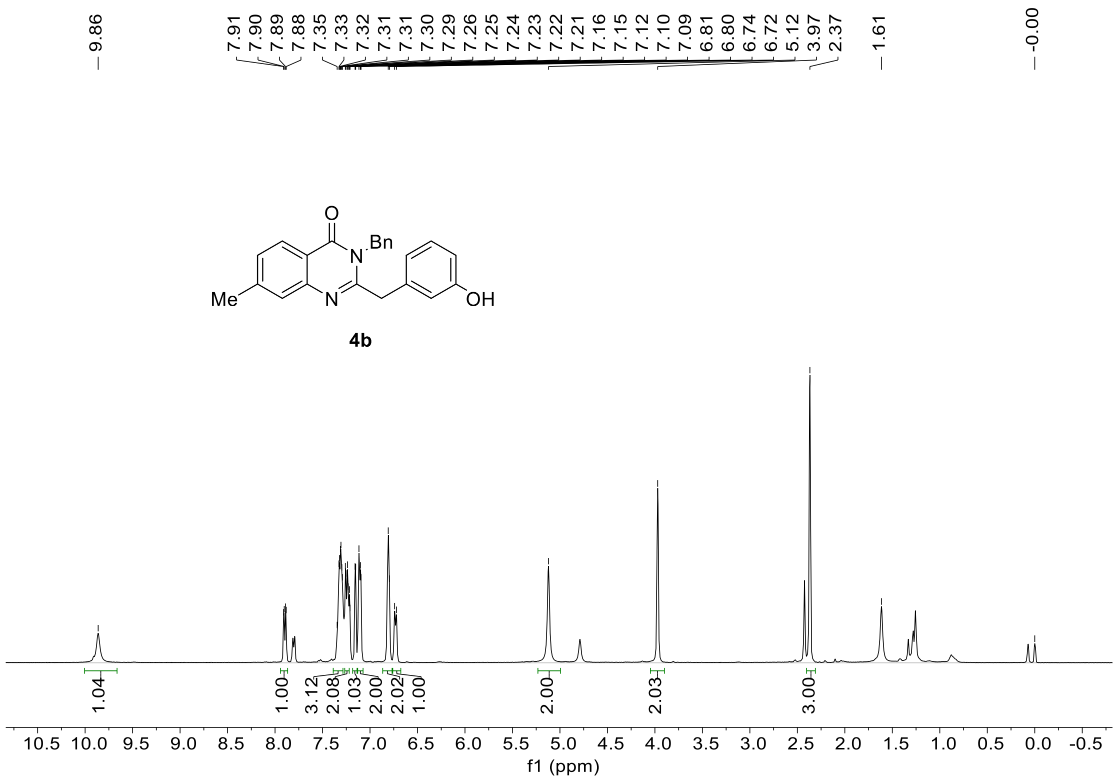

^{13}C NMR spectrum of compound **4b** ($CDCl_3$)

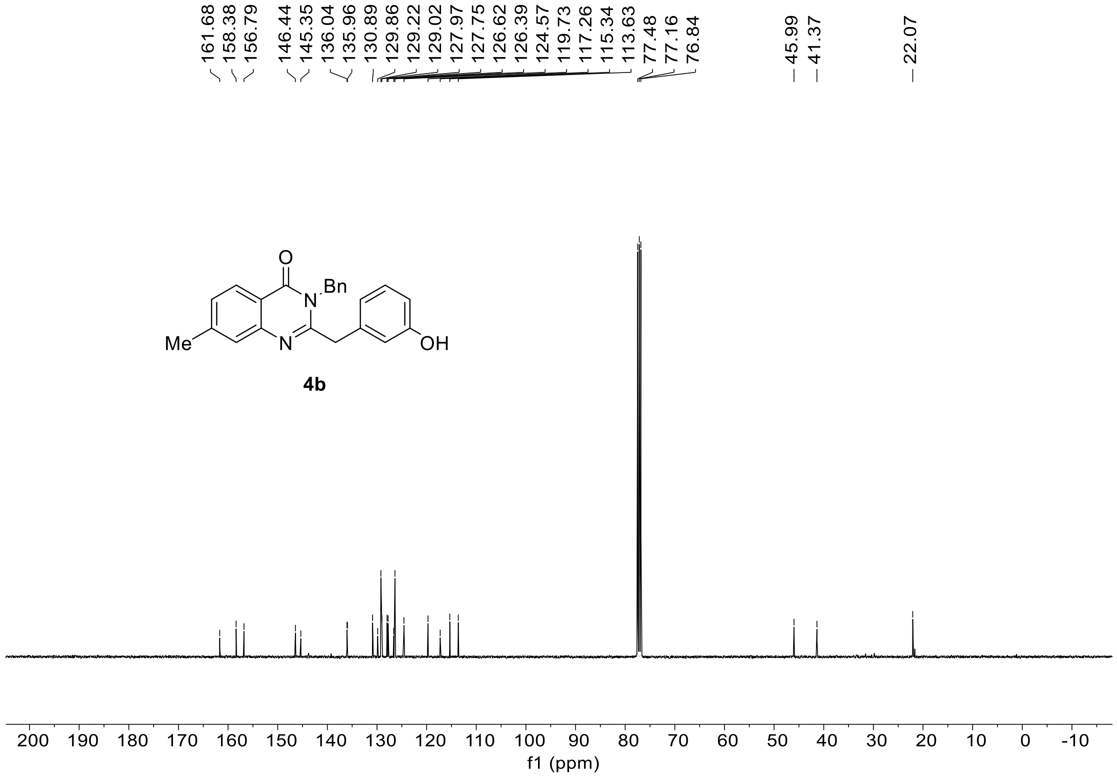

^{1}H NMR spectrum of compound **4c** ($CDCl_3$)

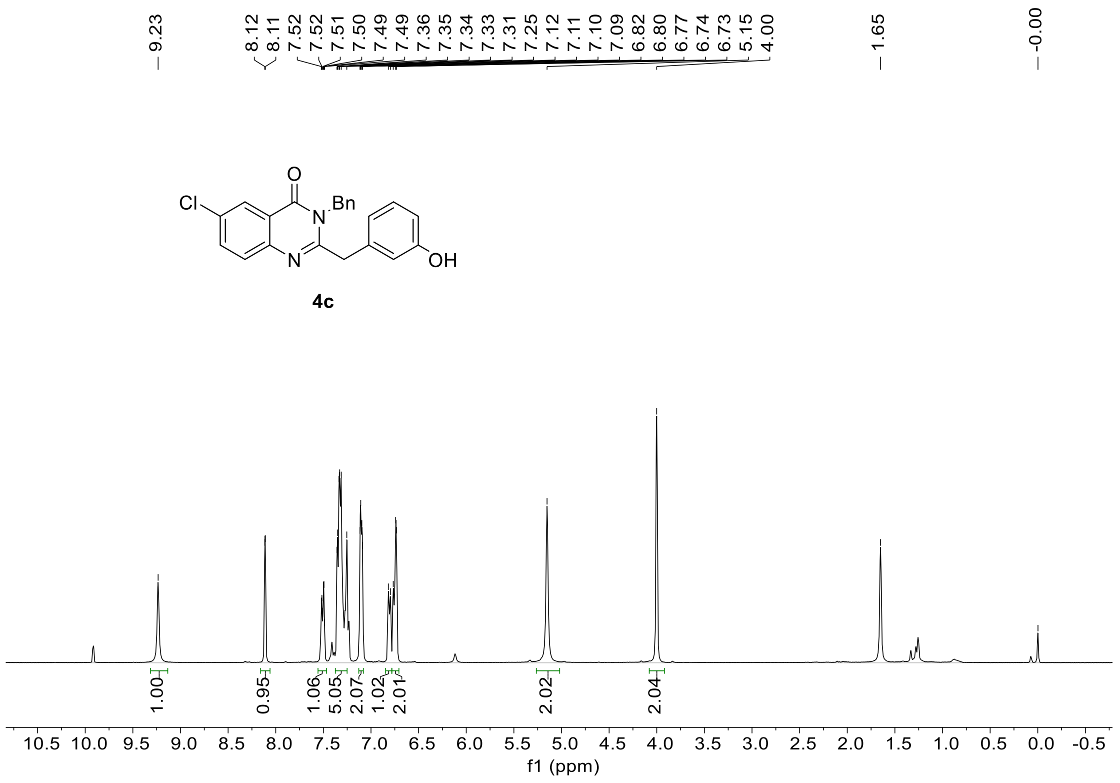

^{13}C NMR spectrum of compound **4c** ($CDCl_3$)

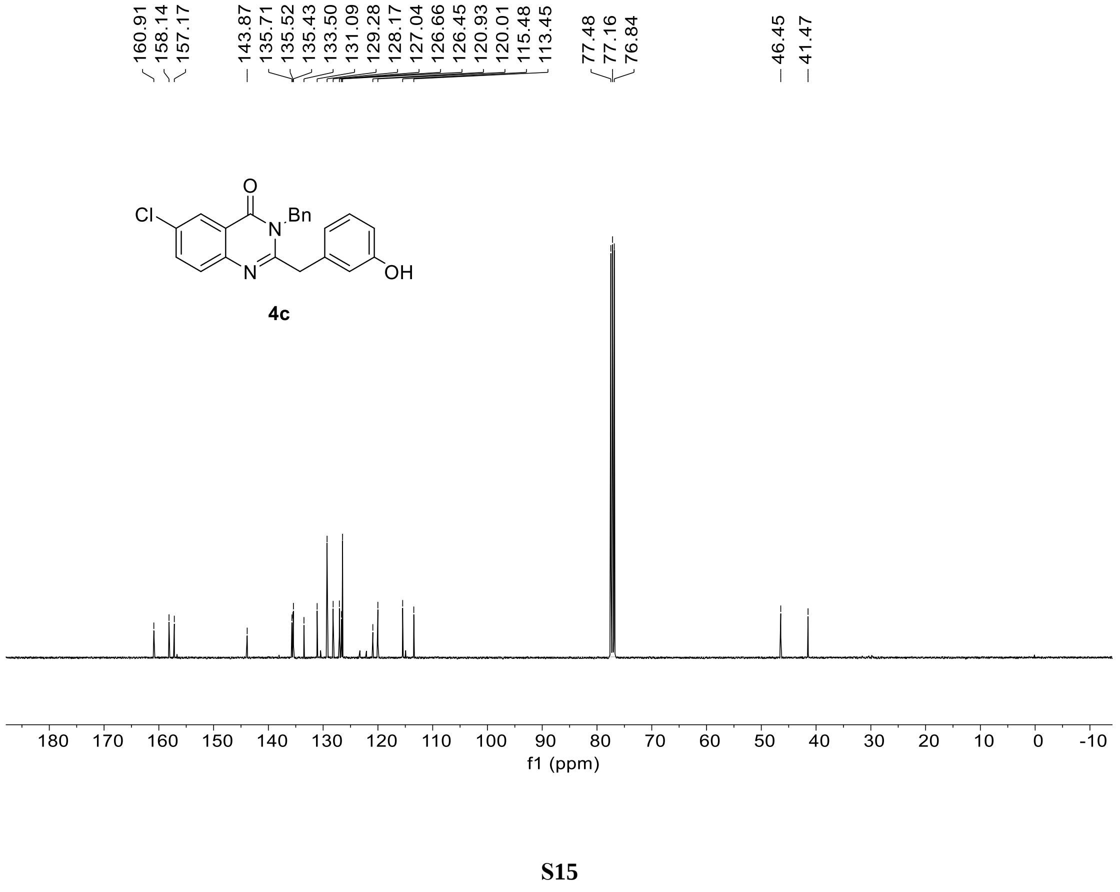

^{1}H NMR spectrum of compound **4d** ($CDCl_3$)

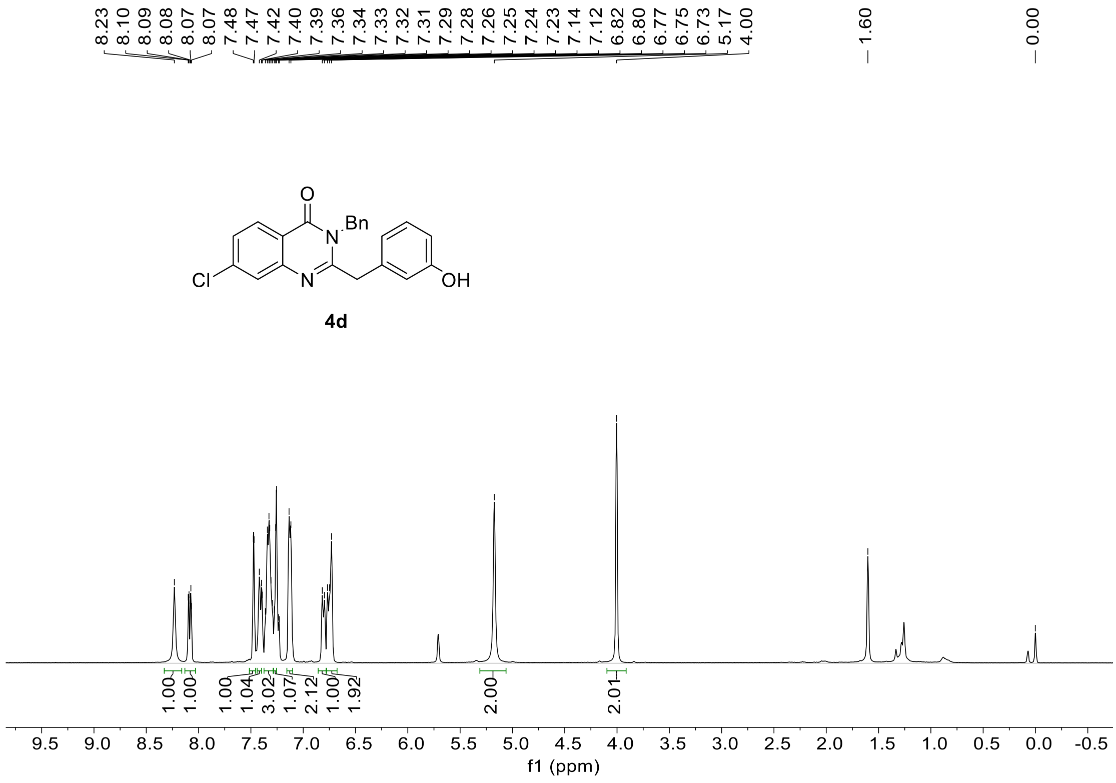

^{13}C NMR spectrum of compound **4d** ($CDCl_3$)

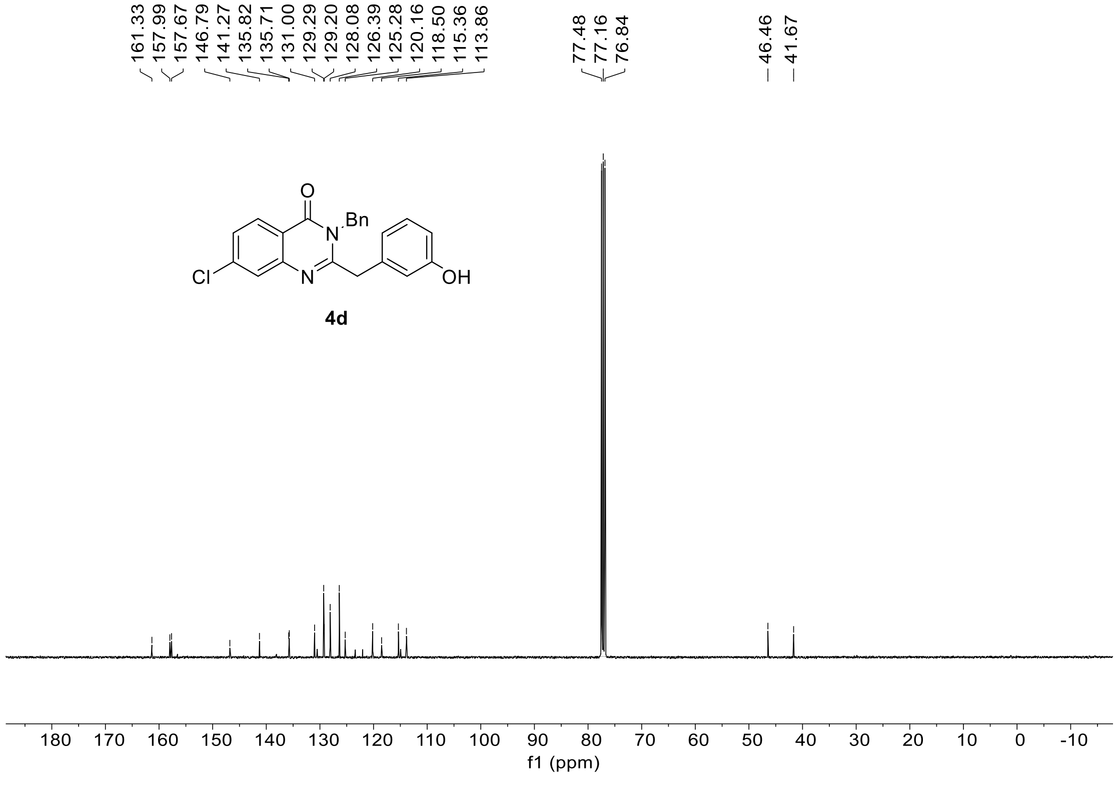

^{1}H NMR spectrum of compound **4e** ($CDCl_3$)

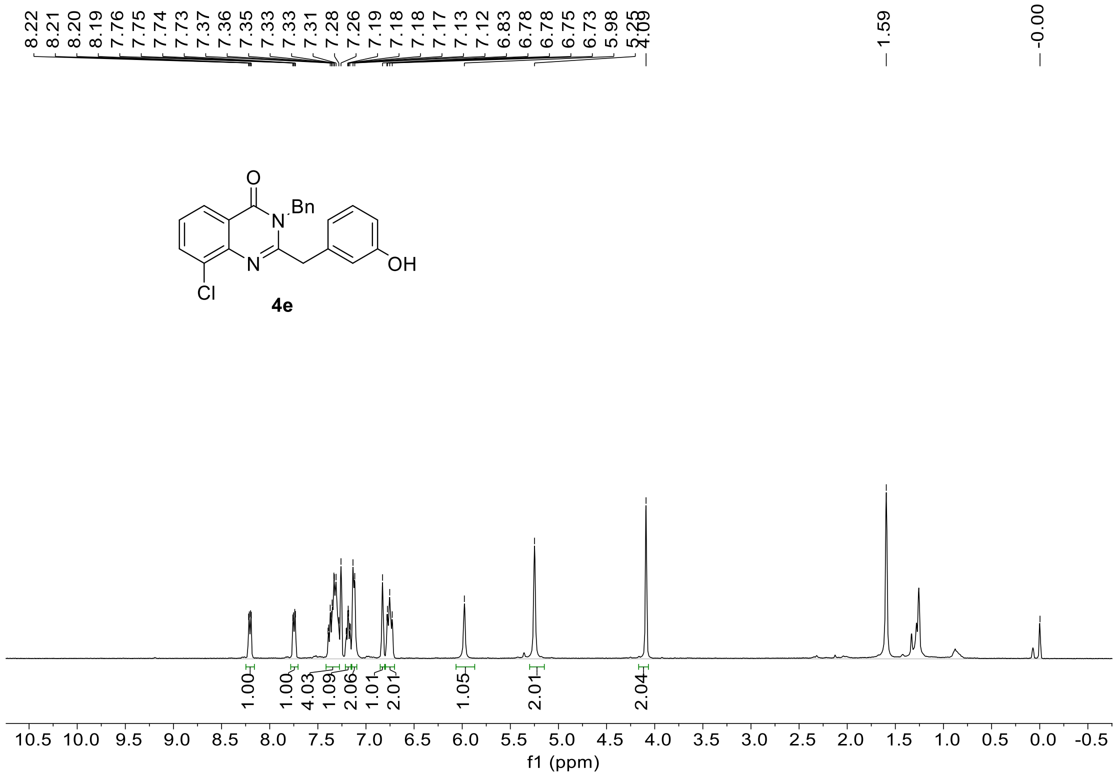

^{13}C NMR spectrum of compound **4e** ($CDCl_3$)

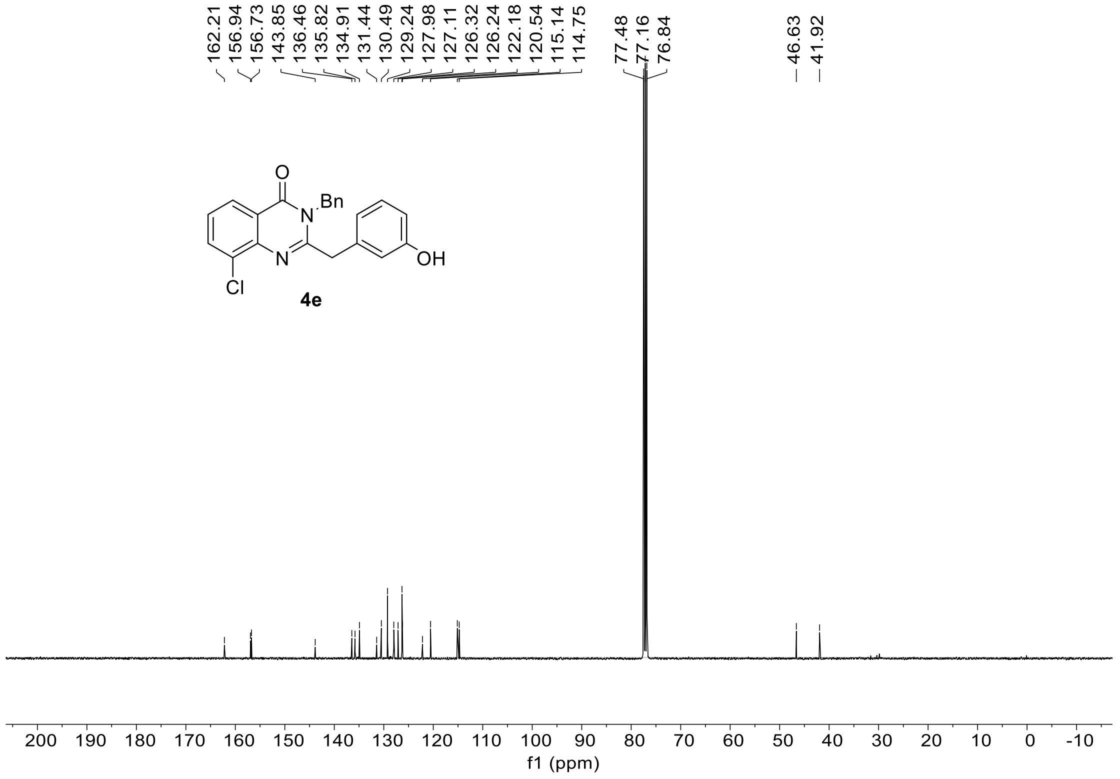

^{1}H NMR spectrum of compound **4f** ($CDCl_3$)

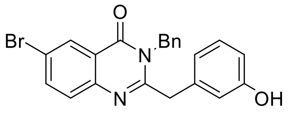

4f

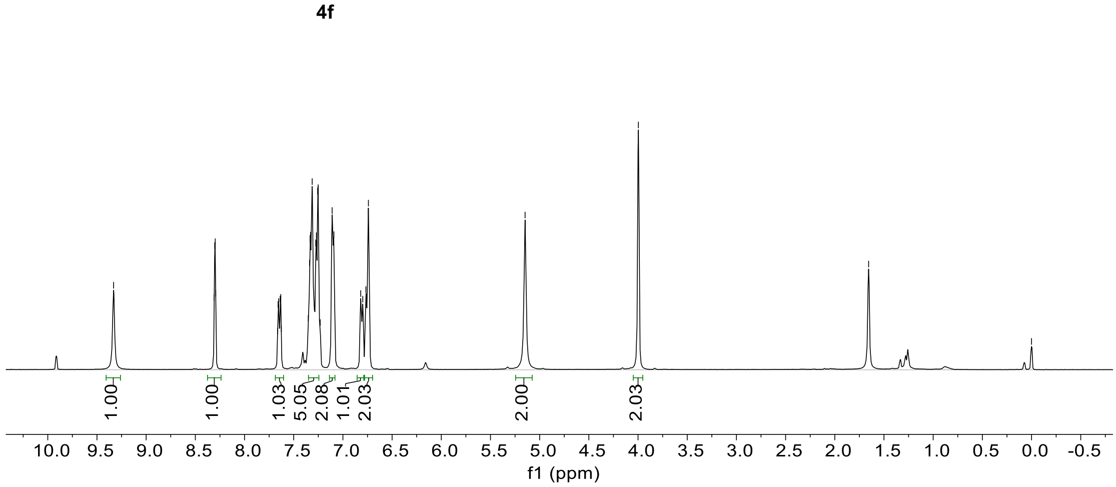

^{13}C NMR spectrum of compound **4f** ($CDCl_3$)

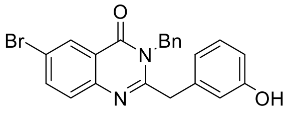

4f

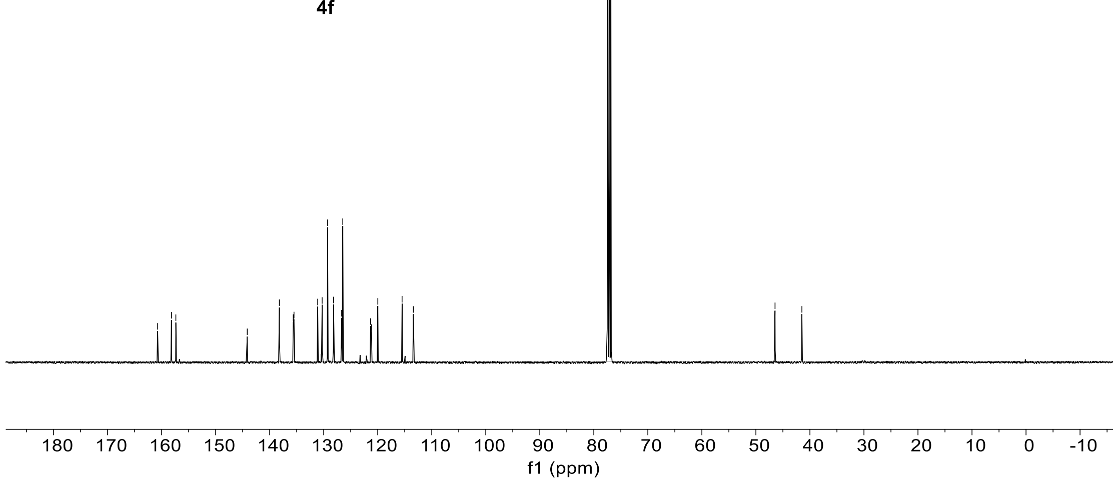

^{1}H NMR spectrum of compound **4g** ($CDCl_3$)

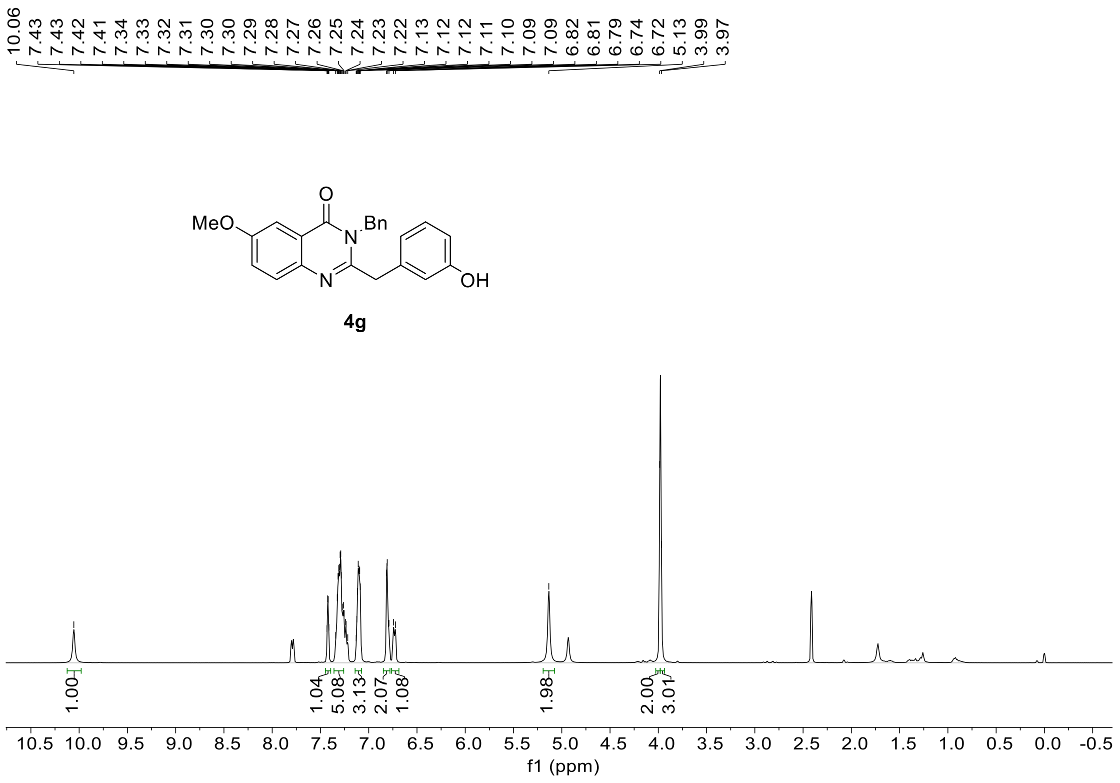

^{13}C NMR spectrum of compound **4g** ($CDCl_3$)

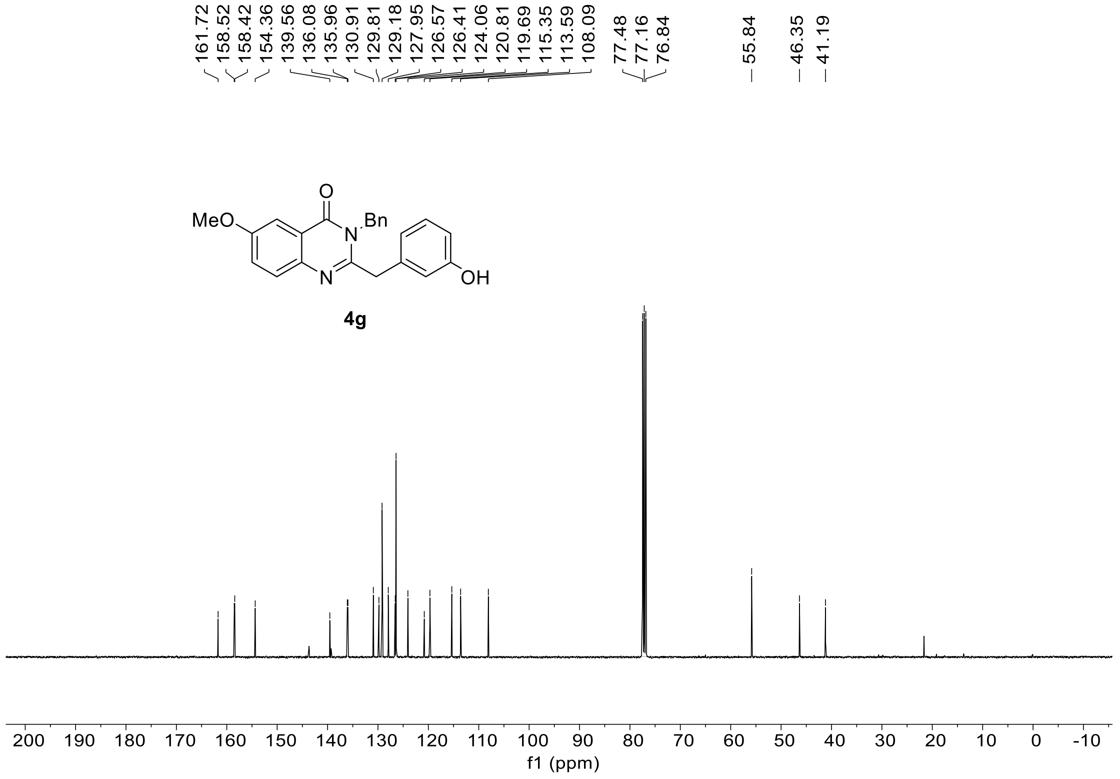

^{1}H NMR spectrum of compound **4j** ($CDCl_3$)

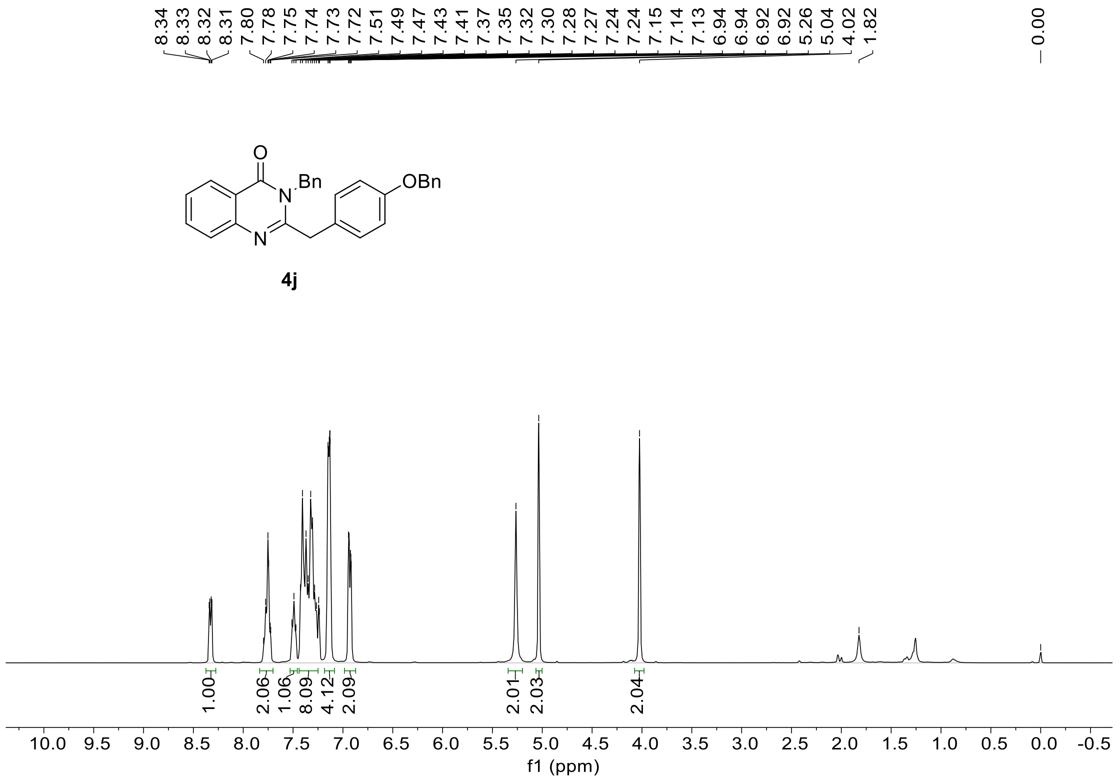

^{13}C NMR spectrum of compound **4j** ($CDCl_3$)

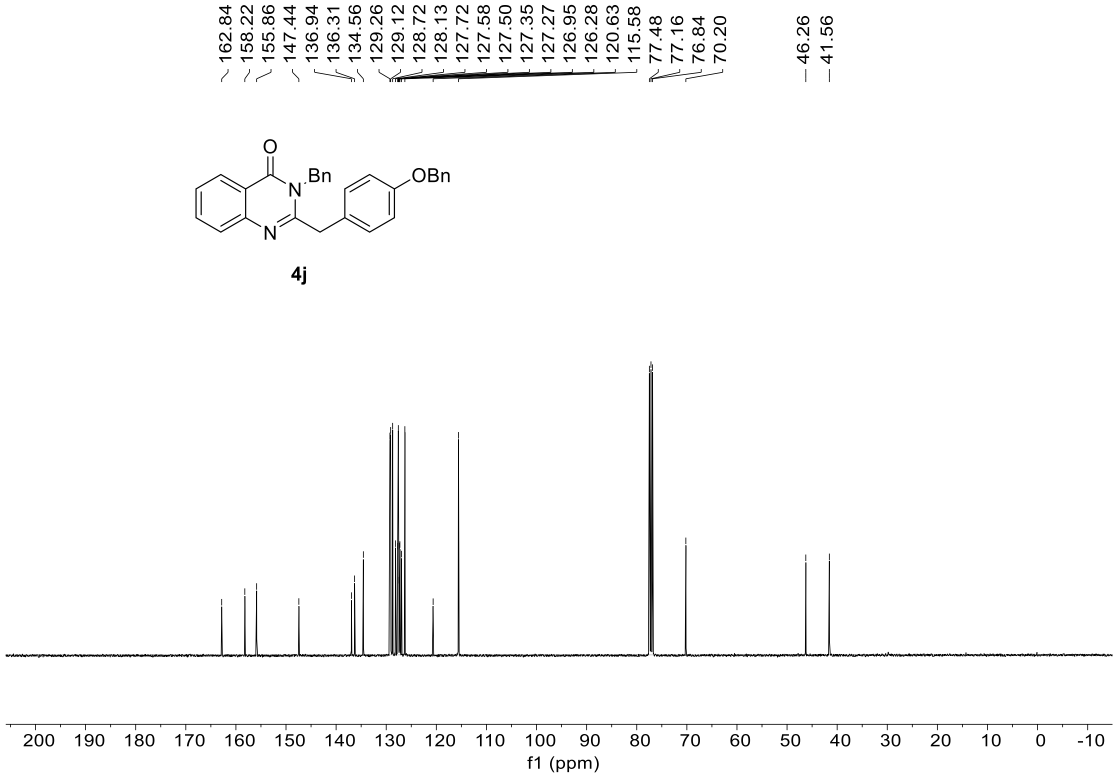

^{1}H NMR spectrum of compound **4k** ($CDCl_3$)

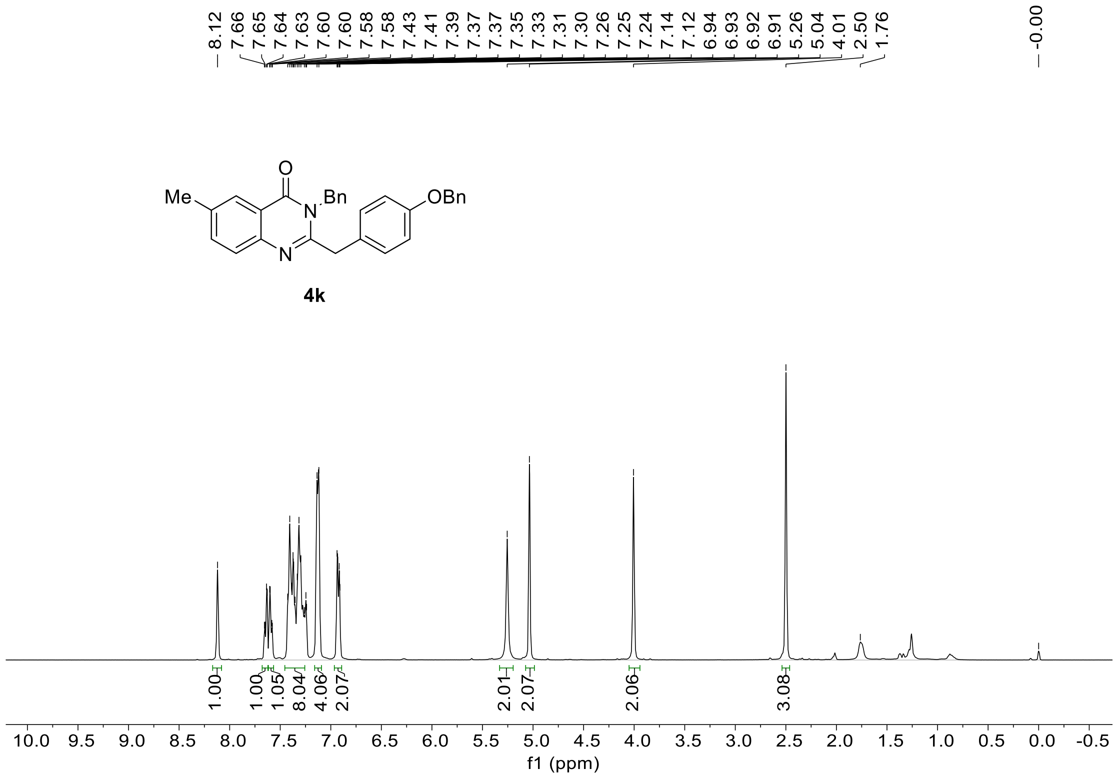

^{13}C NMR spectrum of compound **4k** ($CDCl_3$)

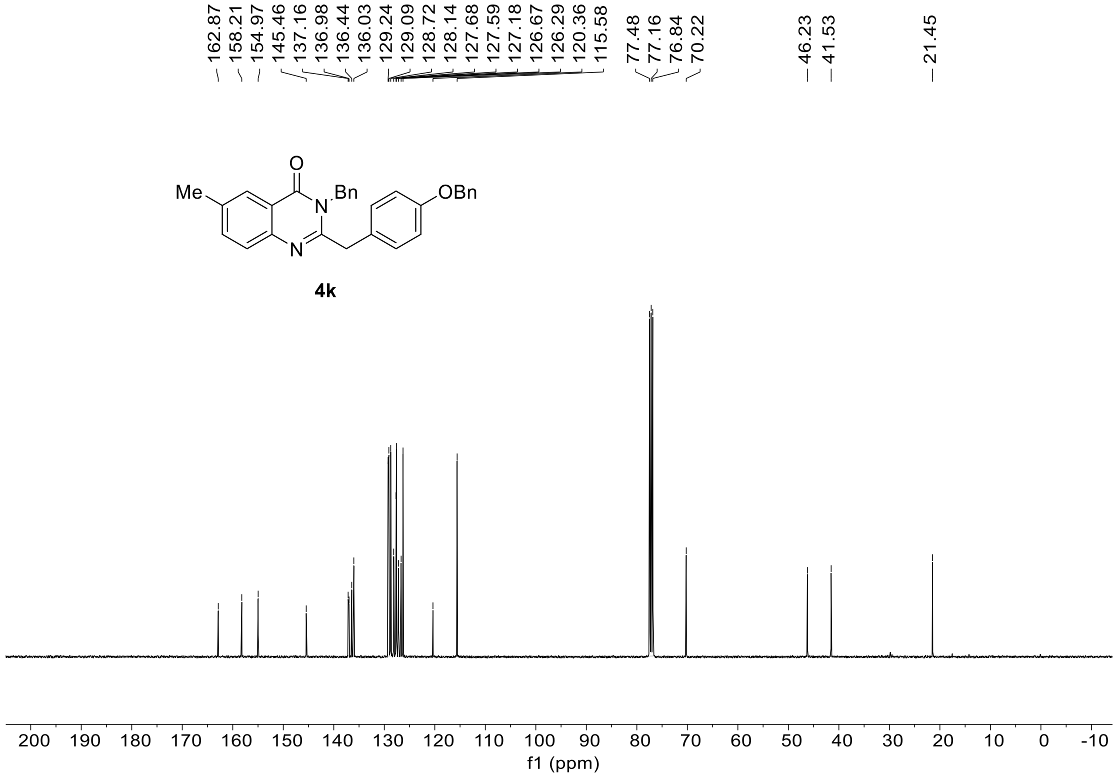

^{1}H NMR spectrum of compound **4l** ($CDCl_3$)

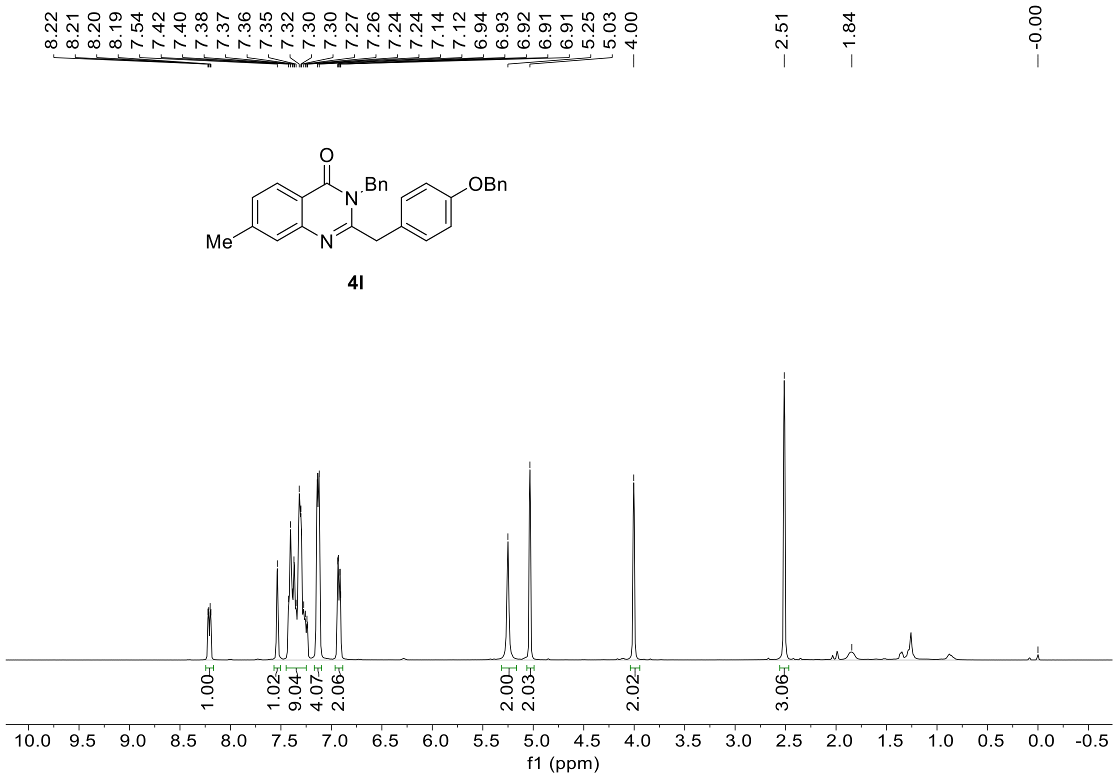

^{13}C NMR spectrum of compound **4l** ($CDCl_3$)

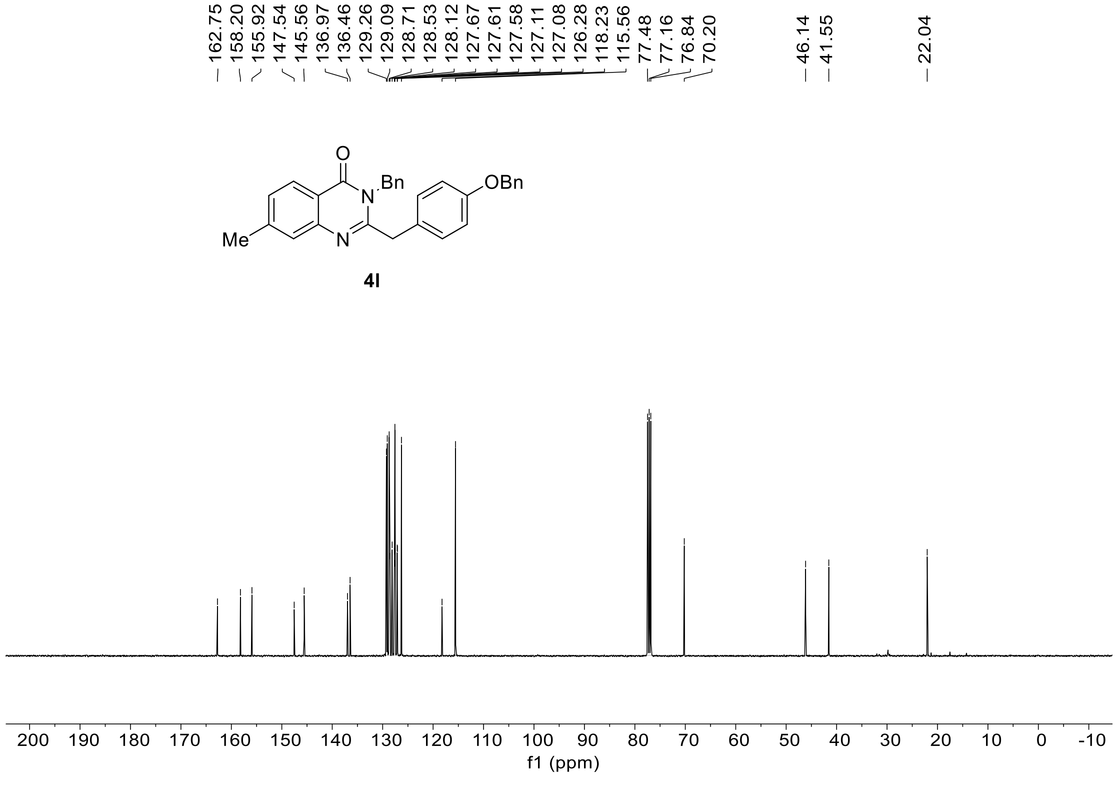

^{1}H NMR spectrum of compound **4m** ($CDCl_3$)

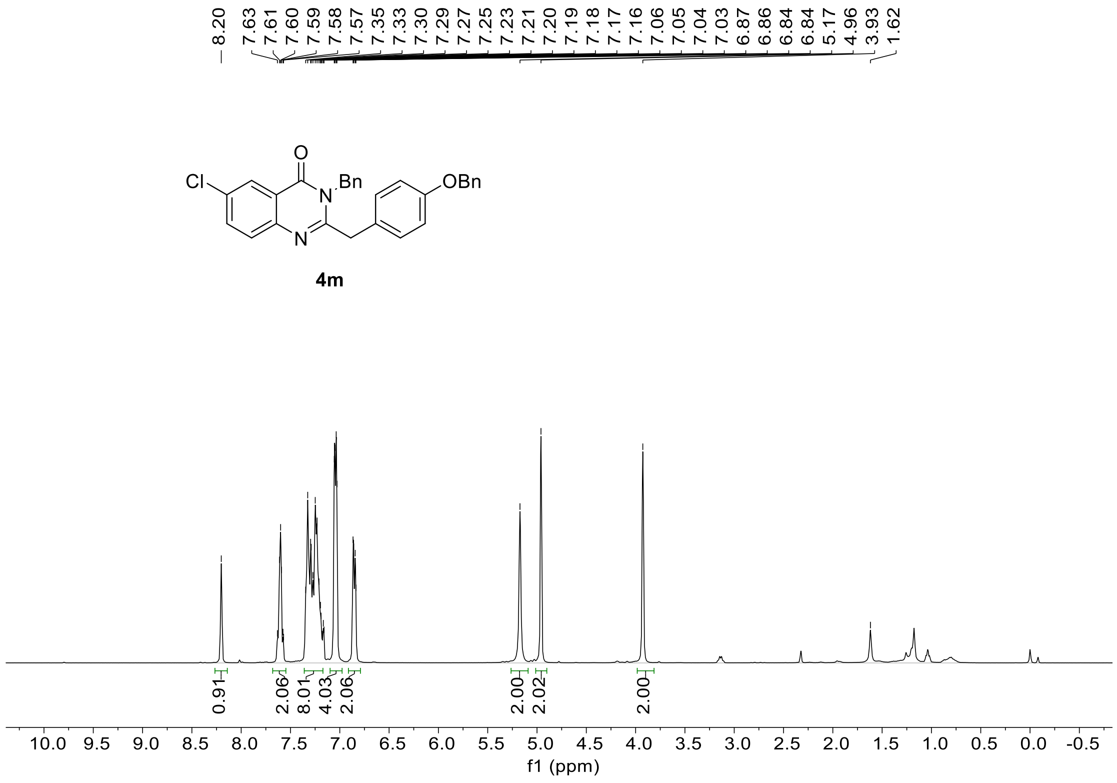

^{13}C NMR spectrum of compound **4m** ($CDCl_3$)

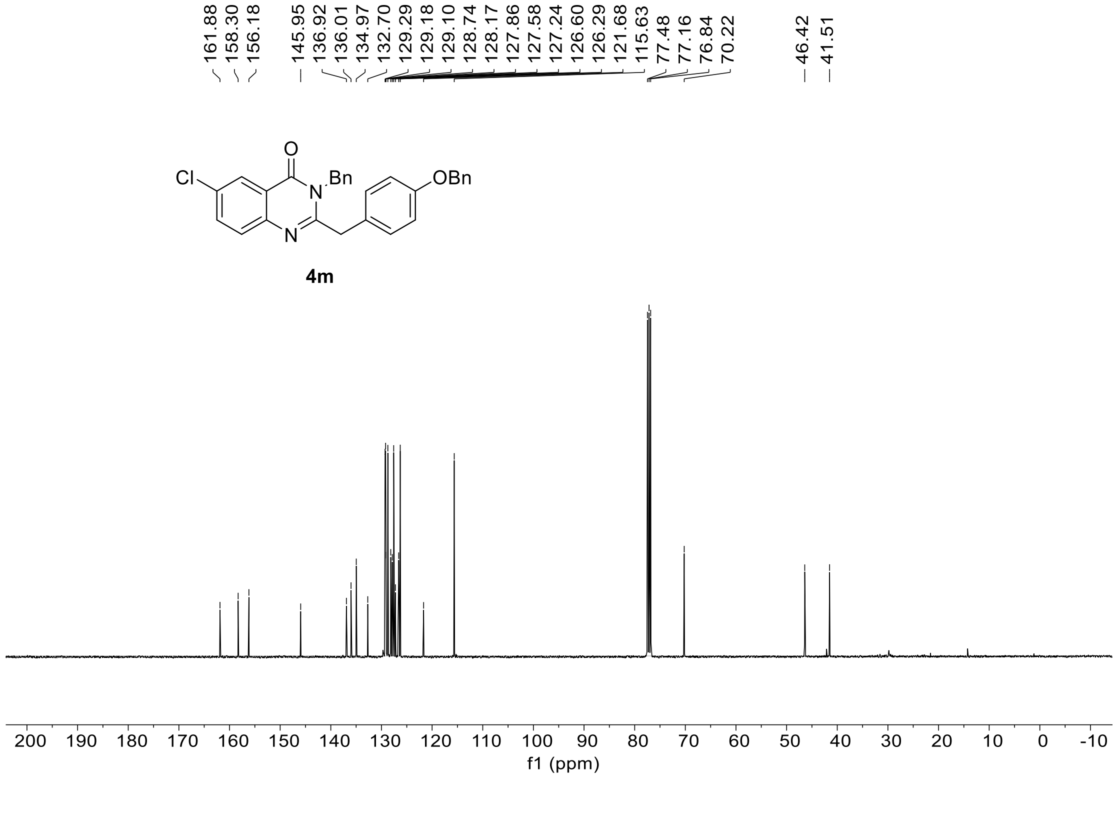

^{1}H NMR spectrum of compound **4n** ($CDCl_3$)

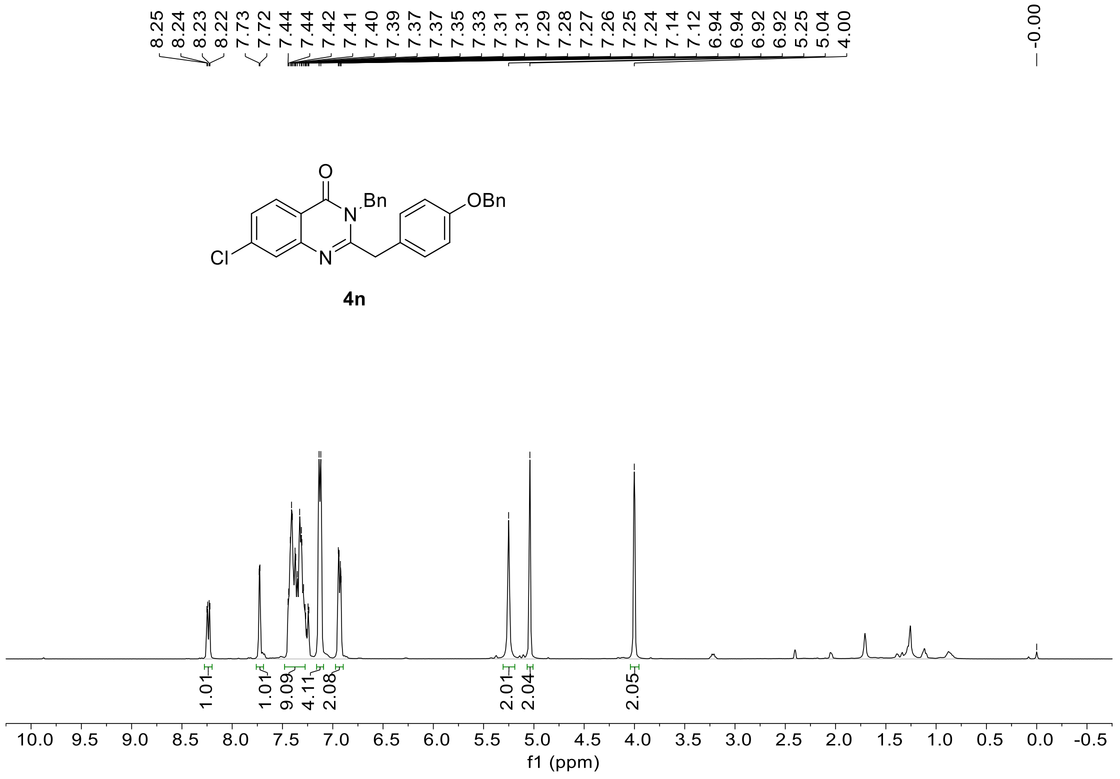

^{13}C NMR spectrum of compound **4n** ($CDCl_3$)

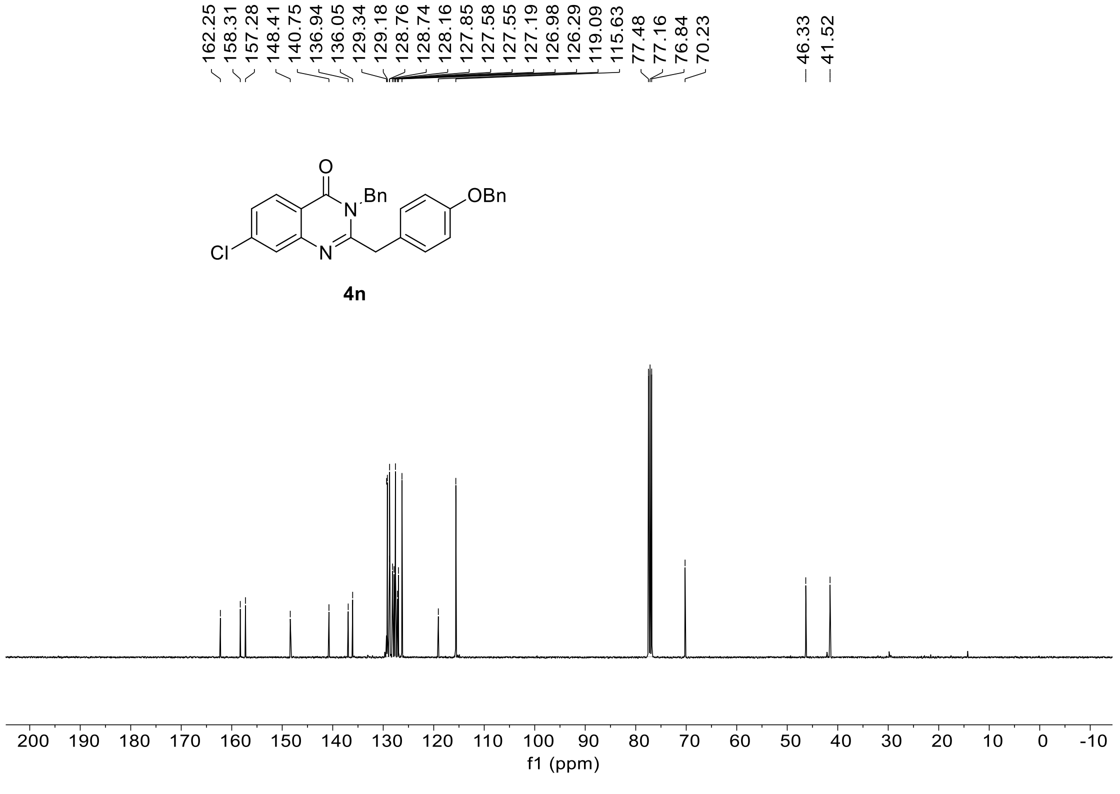

^{1}H NMR spectrum of compound **4o** ($CDCl_3$)

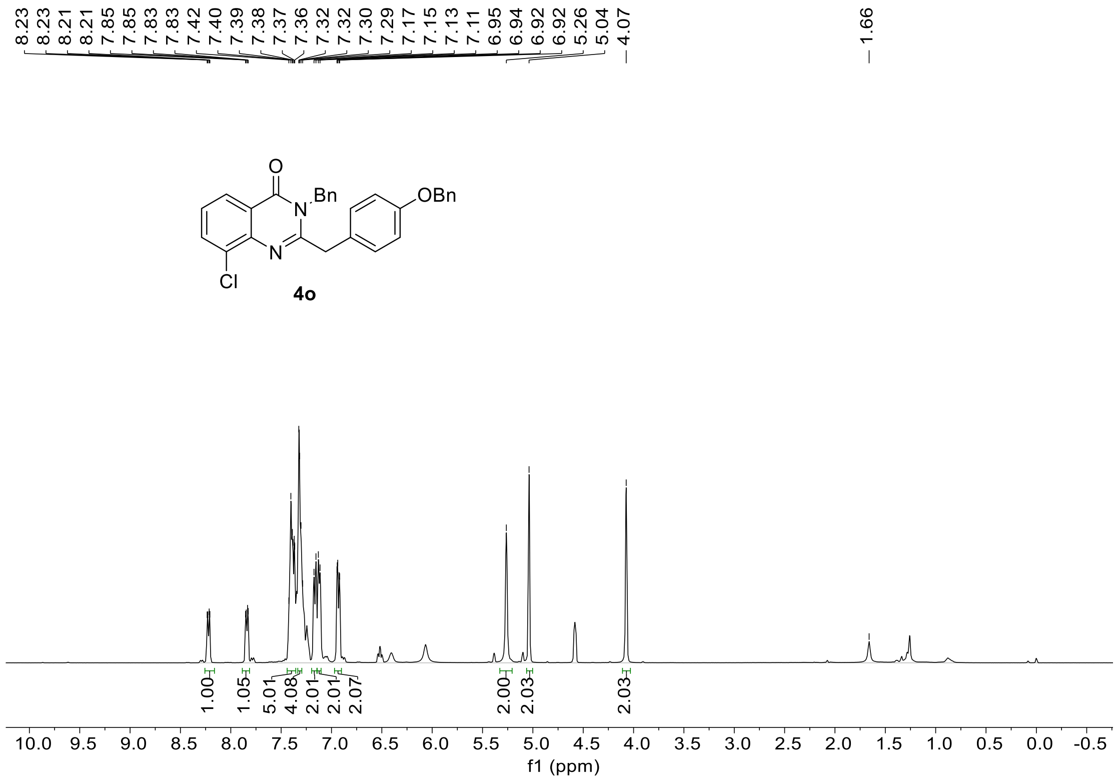

^{13}C NMR spectrum of compound **4o** ($CDCl_3$)

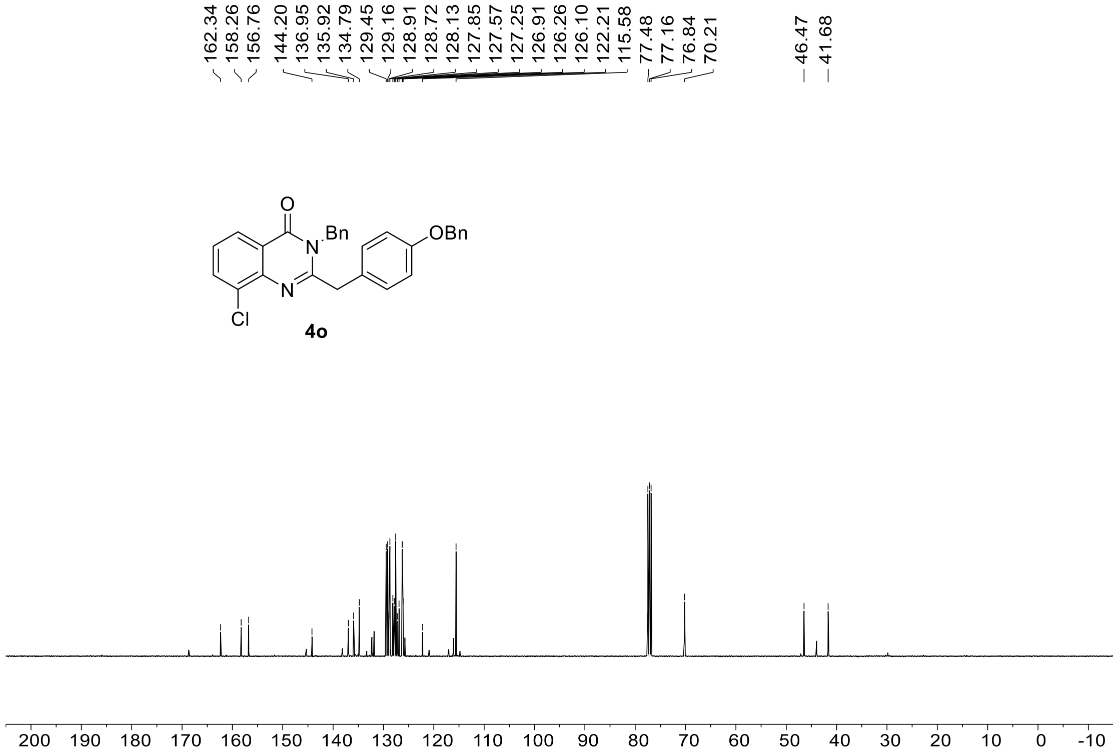

^{1}H NMR spectrum of compound **4p** ($CDCl_3$)

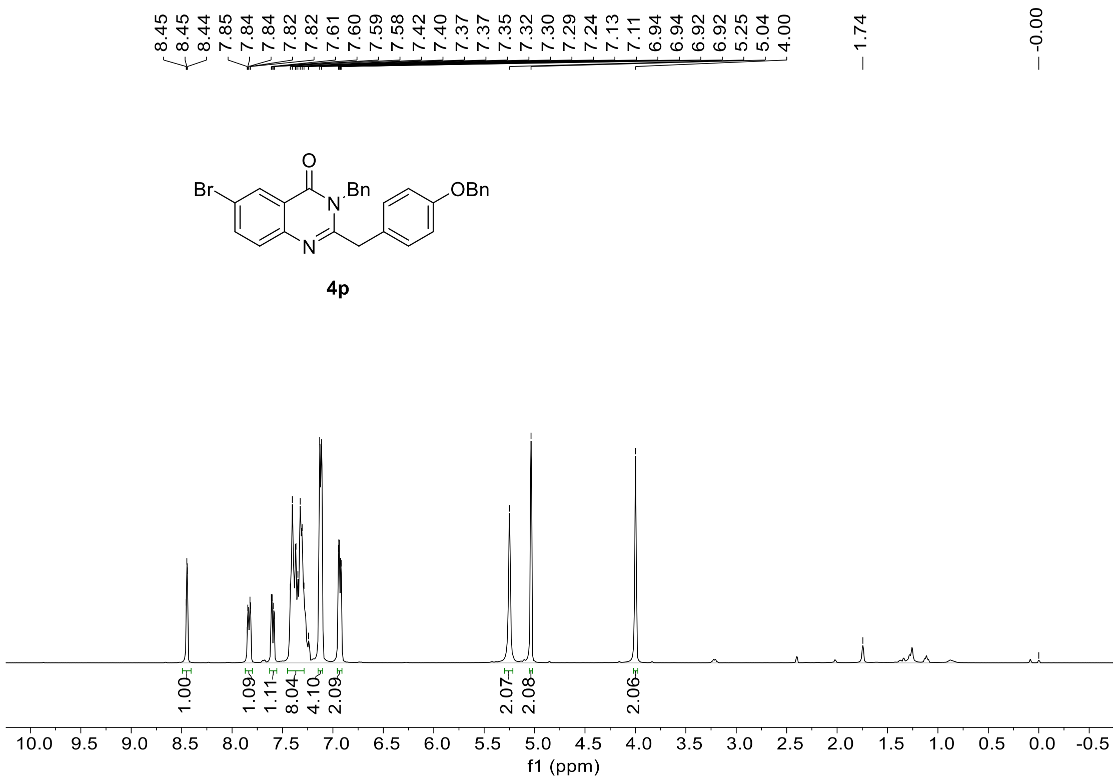

^{13}C NMR spectrum of compound **4p** ($CDCl_3$)

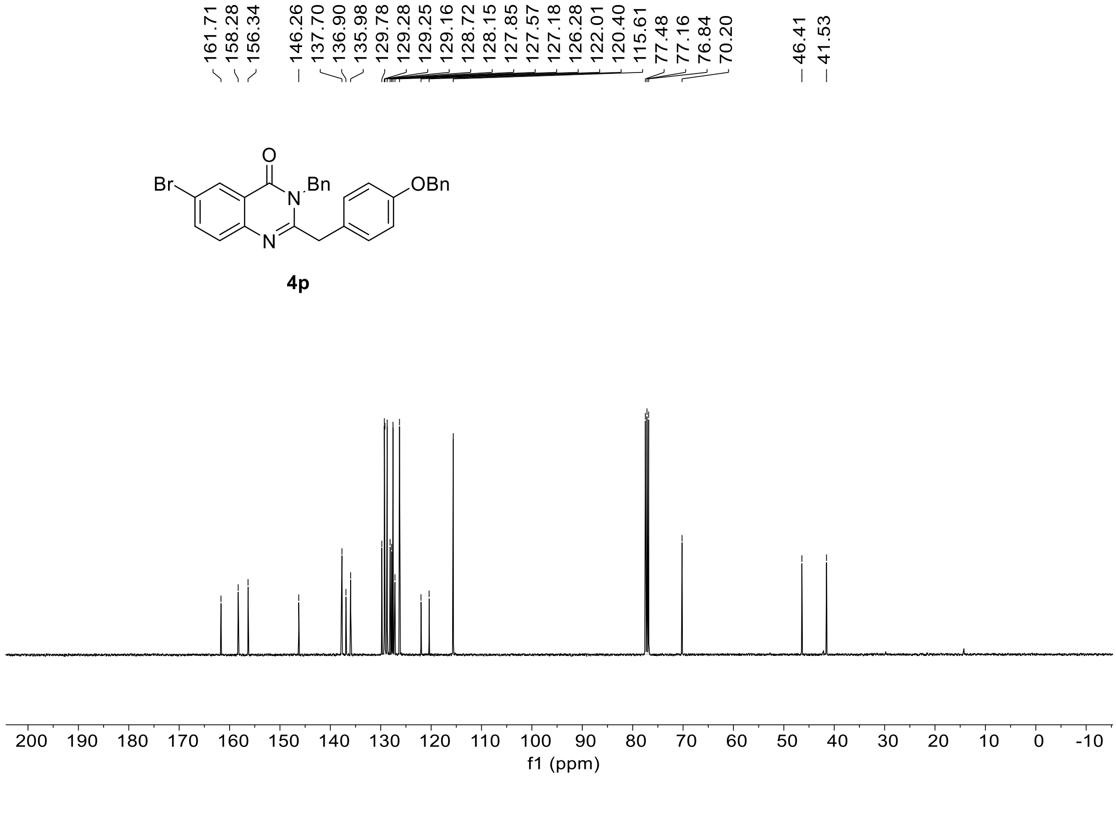

^{1}H NMR spectrum of compound **4q** ($CDCl_3$)

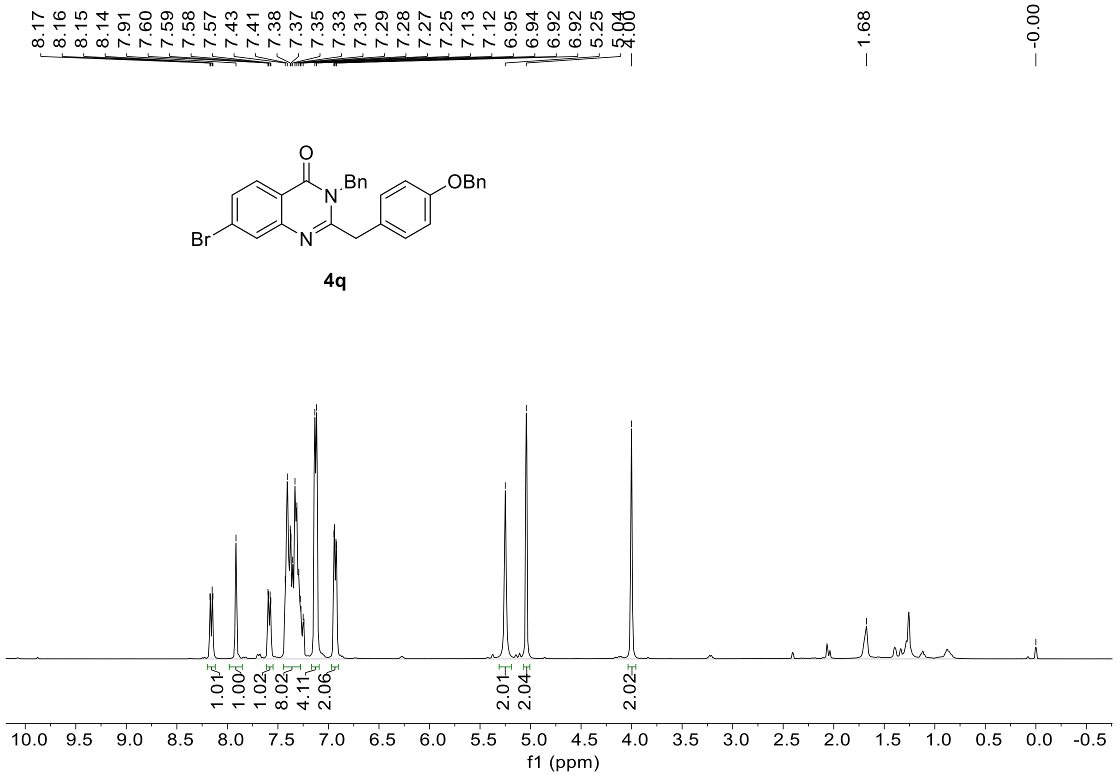

^{13}C NMR spectrum of compound **4q** ($CDCl_3$)

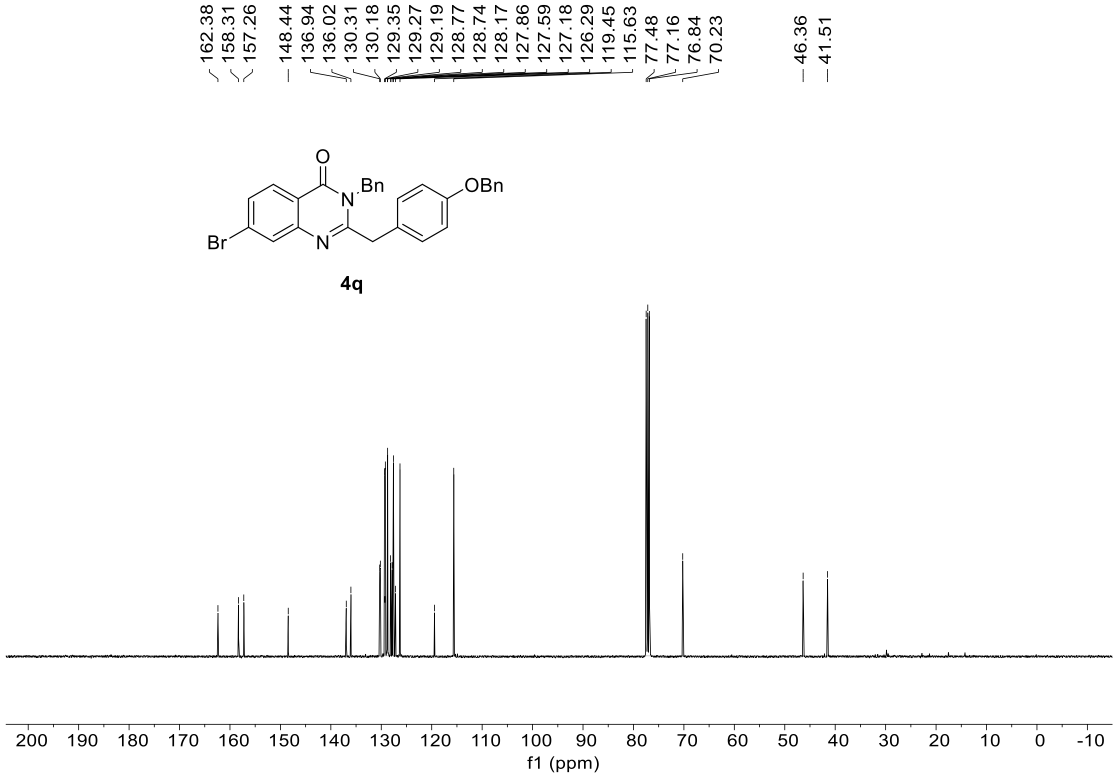

^{1}H NMR spectrum of compound **4r** ($CDCl_3$)

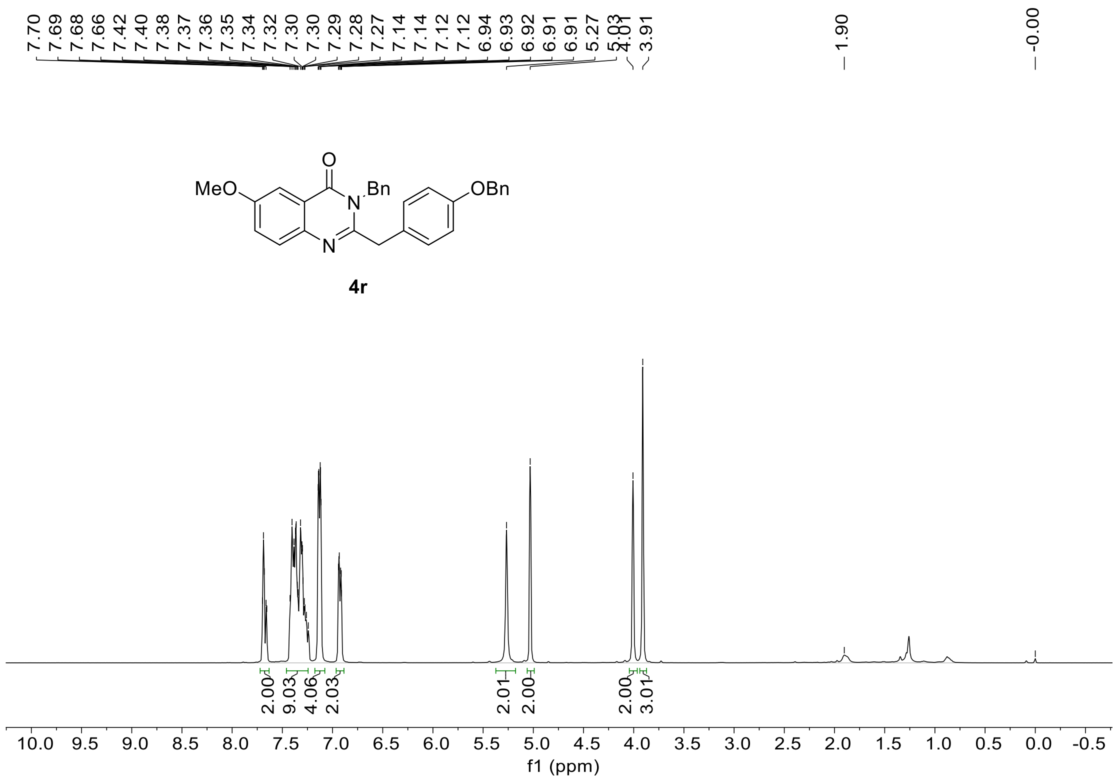

^{13}C NMR spectrum of compound **4r** ($CDCl_3$)

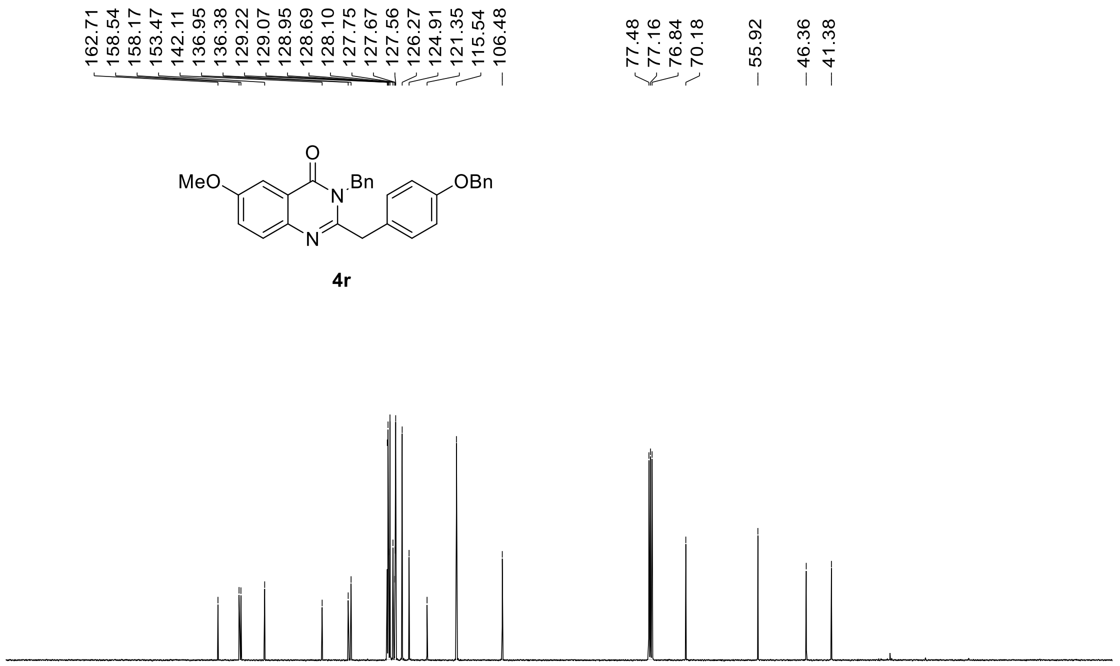

^{1}H NMR spectrum of compound **4s** ($CDCl_3$)

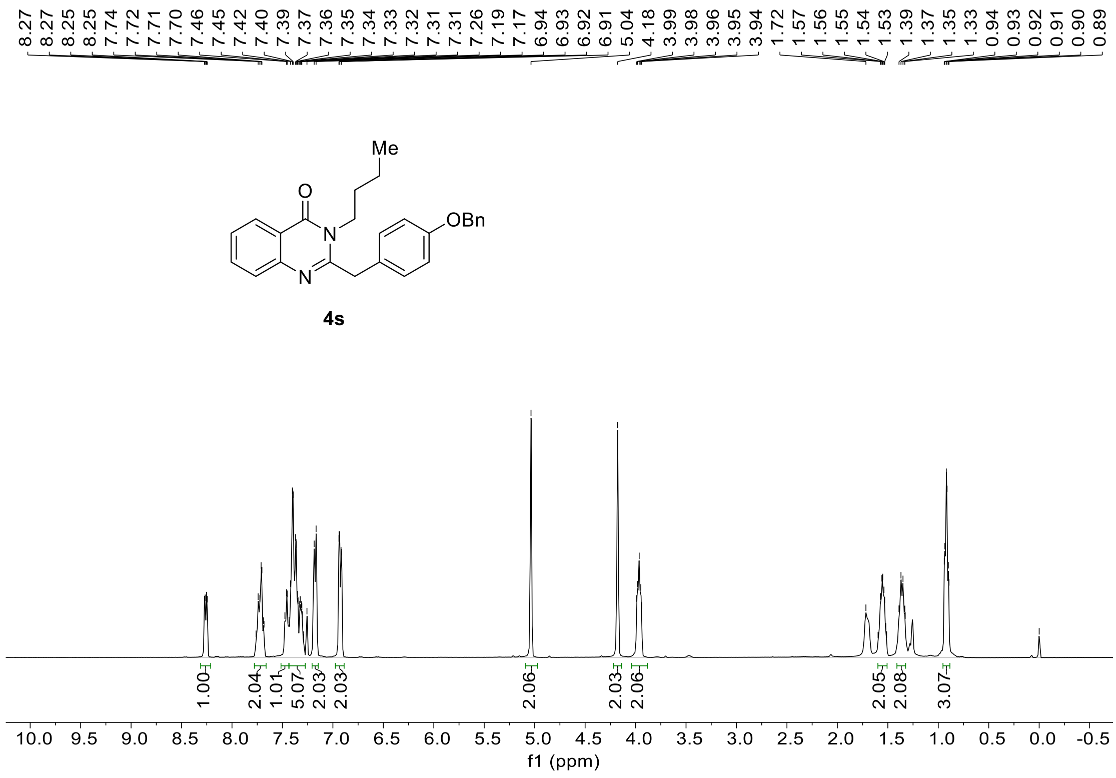

^{13}C NMR spectrum of compound **4s** ($CDCl_3$)

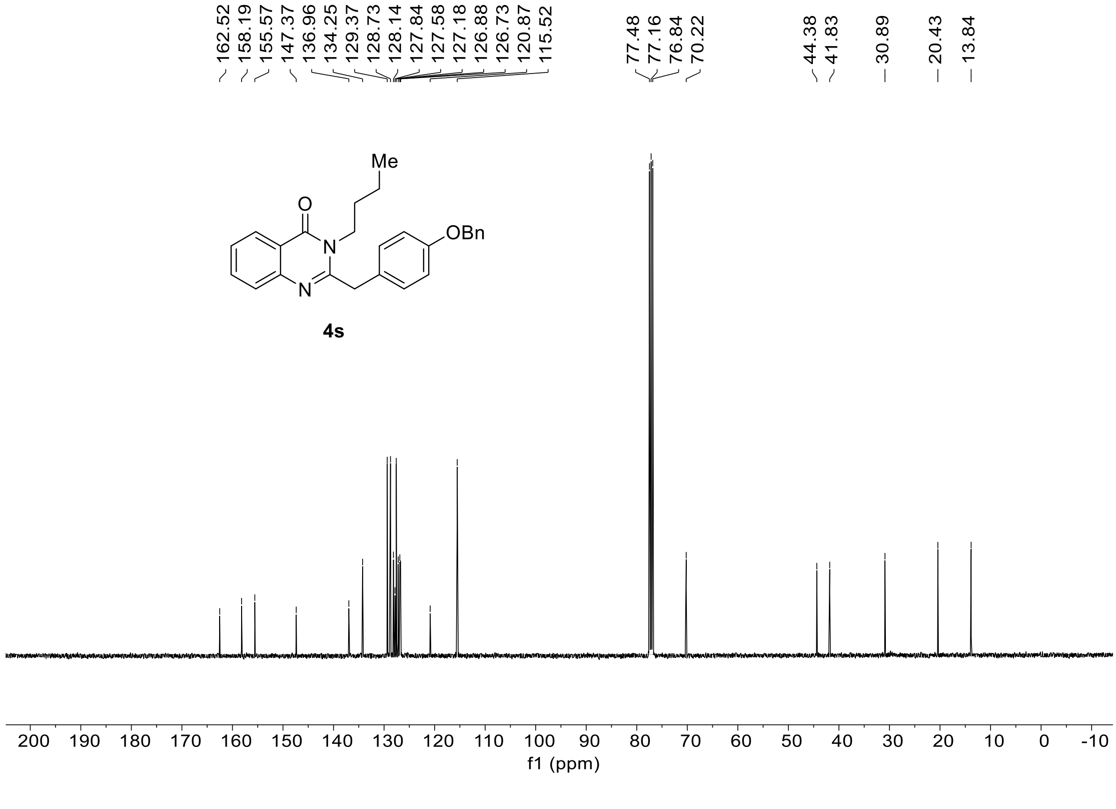

^{1}H NMR spectrum of compound **4t** ($CDCl_3$)

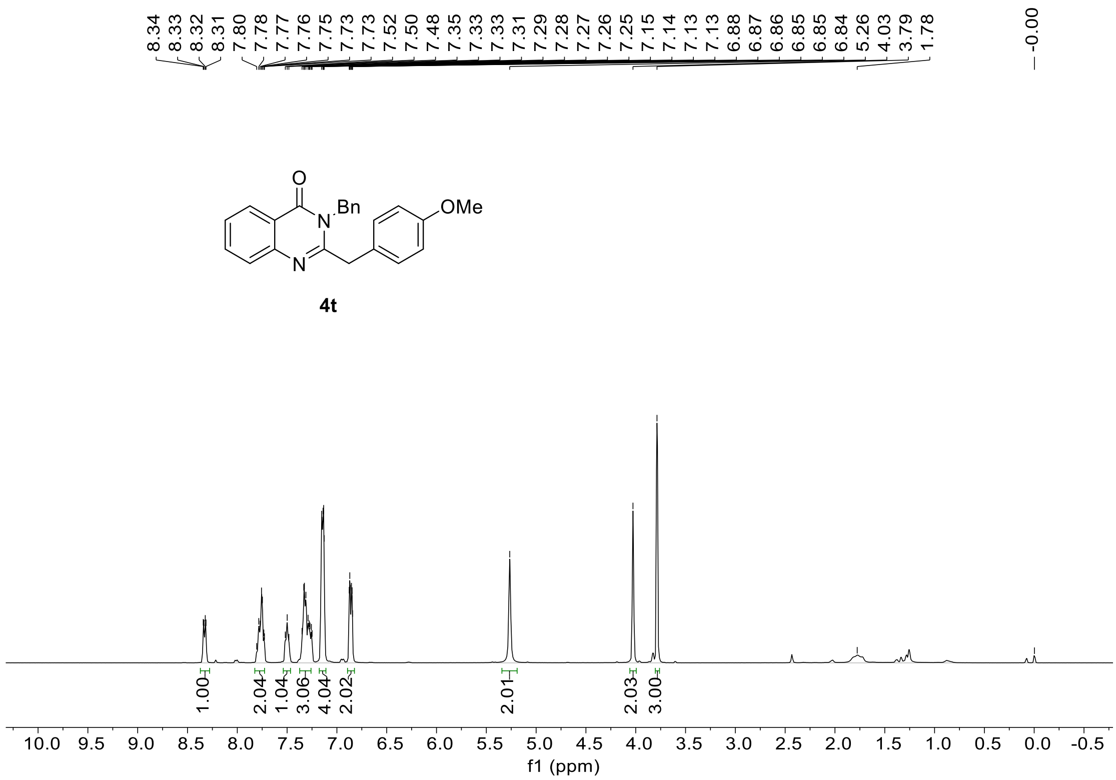

^{13}C NMR spectrum of compound **4t** ($CDCl_3$)

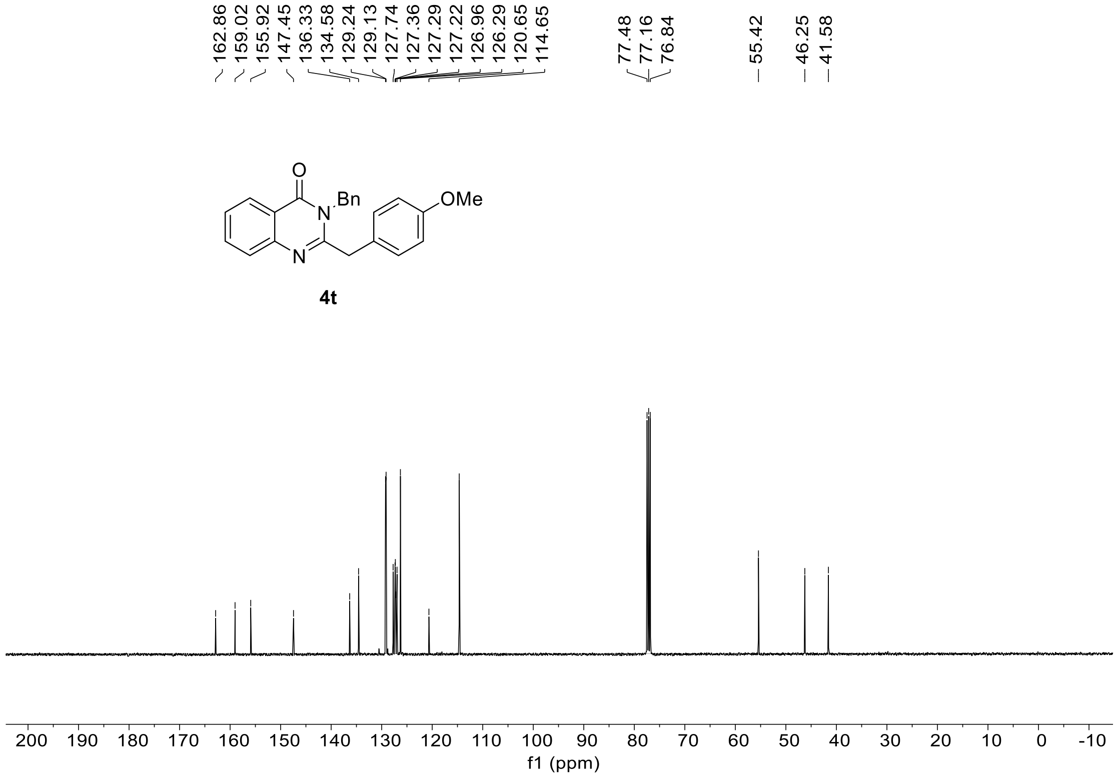

^{1}H NMR spectrum of compound **HBQ** (CD_3OD)

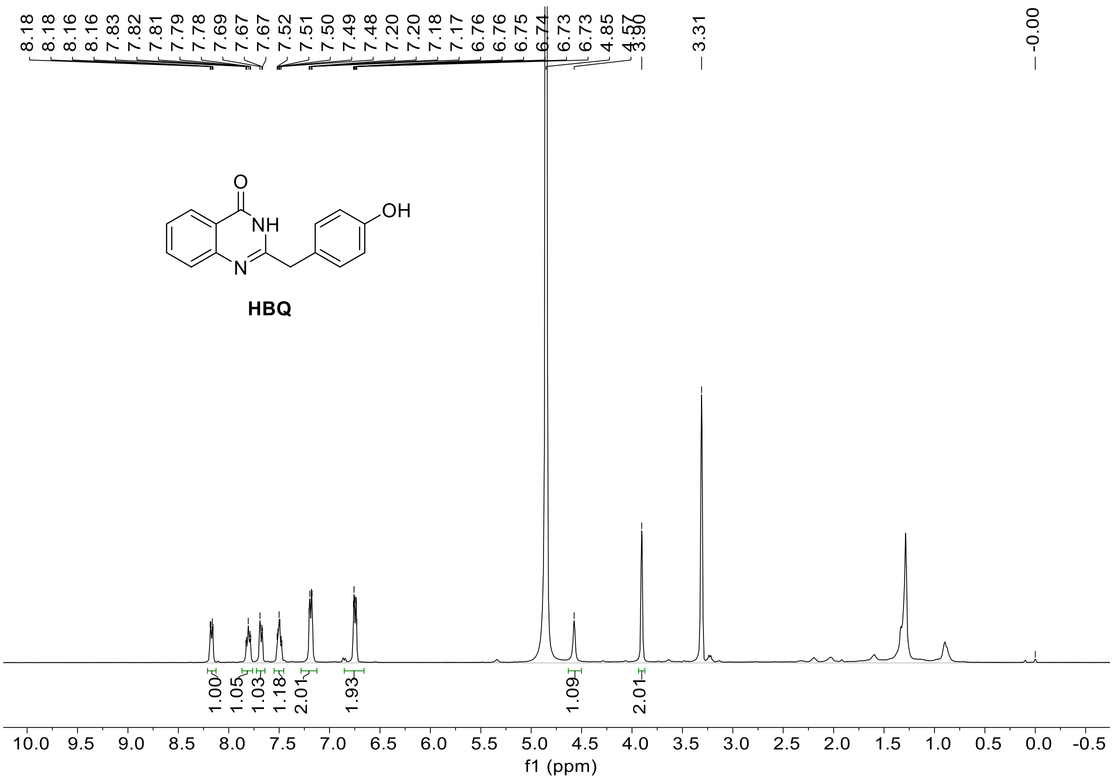

^{13}C NMR spectrum of compound **HBQ** (CD_3OD)

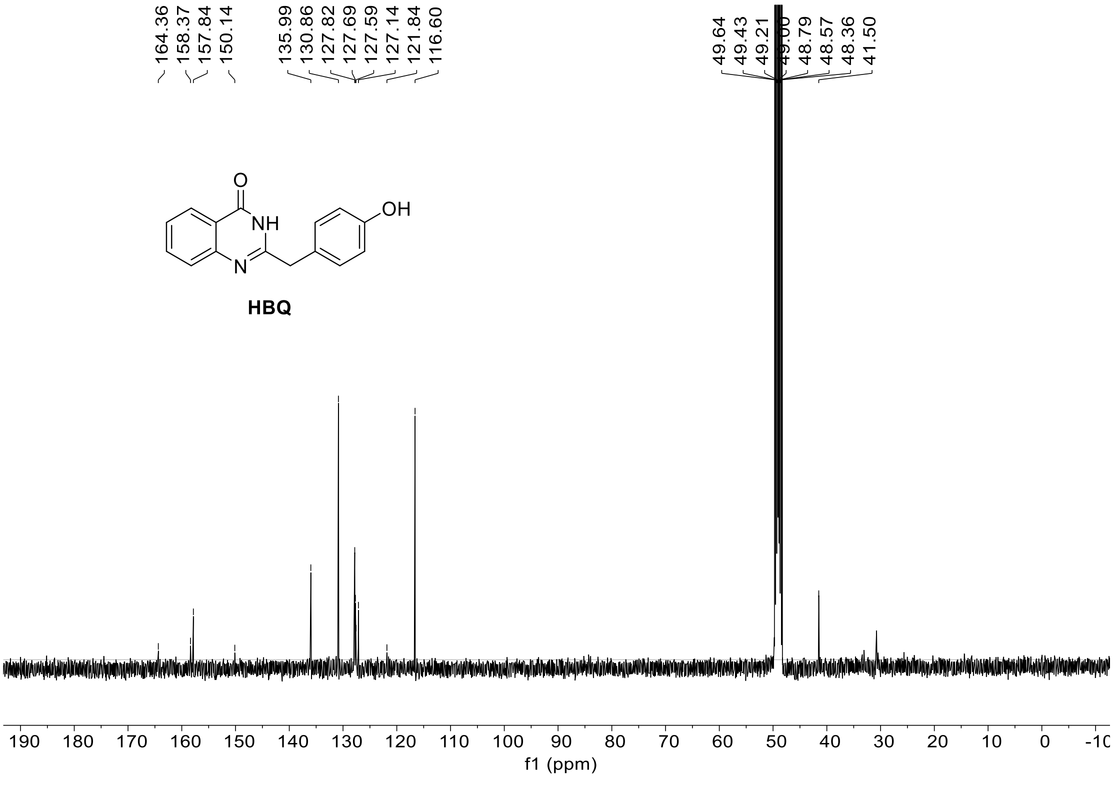

Supplement: Supplementary file 1 [file molecules-28-05734-s001.zip › molecules-2506901-supplementary.pdf]
